# Supplementary material for: Effects of commercial beverages on the neurobehavioral motility of Caenorhabditis elegans
Source: PeerJ. 2022 Jul 14;10:e13563. doi: 10.7717/peerj.13563 (PMC9288823; doi:10.7717/peerj.13563)
Supplement: Supplemental Information 1 [file peerj-10-13563-s001.docx]

**Table S1 Survival rate of nematodes after exposure to beverages**

| **category** | **sample** | **dose(μL/mL)** | **N0** | **N24** | **survival rate(%)** |
| --- | --- | --- | --- | --- | --- |
| Fruit juice | mixed juice | 500 | 60 | 60 | 100 |
|  |  | 250 | 60 | 60 | 100 |
|  |  | 125 | 60 | 60 | 100 |
|  |  | 62.5 | 60 | 60 | 100 |
|  | Single juice | 500 | 60 | 60 | 100 |
|  |  | 250 | 60 | 60 | 100 |
|  |  | 125 | 60 | 60 | 100 |
|  |  | 62.5 | 60 | 60 | 100 |
| Carbonated drinks | Brown carbonated beverage | 500 | 60 | 60 | 100 |
|  |  | 250 | 60 | 60 | 100 |
|  |  | 125 | 60 | 60 | 100 |
|  |  | 62.5 | 60 | 60 | 100 |
|  | Colorless carbonated beverage | 500 | 60 | 60 | 100 |
|  |  | 250 | 60 | 60 | 100 |
|  |  | 125 | 60 | 60 | 100 |
|  |  | 62.5 | 60 | 60 | 100 |
|  | Orange carbonated beverage | 500 | 60 | 60 | 100 |
|  |  | 250 | 60 | 60 | 100 |
|  |  | 125 | 60 | 60 | 100 |
|  |  | 62.5 | 60 | 60 | 100 |
| Functional beverage | Sports functional drink | 500 | 60 | 60 | 100 |
|  |  | 250 | 60 | 60 | 100 |
|  |  | 125 | 60 | 60 | 100 |
|  |  | 62.5 | 60 | 60 | 100 |
|  | Fatigue relieving functional drink | 500 | 60 | 60 | 100 |
|  |  | 250 | 60 | 60 | 100 |
|  |  | 125 | 60 | 60 | 100 |
|  |  | 62.5 | 60 | 60 | 100 |
| Tea beverage | Black tea beverage | 500 | 60 | 60 | 100 |
|  |  | 250 | 60 | 60 | 100 |
|  |  | 125 | 60 | 60 | 100 |
|  |  | 62.5 | 60 | 60 | 100 |
|  | Green tea beverage | 500 | 60 | 60 | 100 |
|  |  | 250 | 60 | 60 | 100 |
|  |  | 125 | 60 | 60 | 100 |
|  |  | 62.5 | 60 | 60 | 100 |
|  | Herbal tea drink | 500 | 60 | 60 | 100 |
|  |  | 250 | 60 | 60 | 100 |
|  |  | 125 | 60 | 60 | 100 |
|  |  | 62.5 | 60 | 60 | 100 |

**Continued Table S1 Survival rate of nematodes after exposure to beverages**

| **category** | **sample** | **dose(μL/mL)** | **N0** | **N24** | **survival rate(%)** |
| --- | --- | --- | --- | --- | --- |
| Coffee beverage | Coffee drinks | 500 | 60 | 60 | 100 |
|  |  | 250 | 60 | 60 | 100 |
|  |  | 125 | 60 | 60 | 100 |
|  |  | 62.5 | 60 | 60 | 100 |
| Phytoprotein beverage | Almond milk | 500 | 60 | 60 | 100 |
|  |  | 250 | 60 | 60 | 100 |
|  |  | 125 | 60 | 60 | 100 |
|  |  | 62.5 | 60 | 60 | 100 |
|  | Coconut drink | 500 | 60 | 60 | 100 |
|  |  | 250 | 60 | 60 | 100 |
|  |  | 125 | 60 | 60 | 100 |
|  |  | 62.5 | 60 | 60 | 100 |
|  | Milk tea beverage | 500 | 60 | 60 | 100 |
|  |  | 250 | 60 | 60 | 100 |
|  |  | 125 | 60 | 60 | 100 |
|  |  | 62.5 | 60 | 60 | 100 |
| Dairy products | Prepared milk beverage A | 500 | 60 | 60 | 100 |
|  |  | 250 | 60 | 60 | 100 |
|  |  | 125 | 60 | 60 | 100 |
|  |  | 62.5 | 60 | 60 | 100 |
|  | Prepared milk beverage B | 500 | 60 | 60 | 100 |
|  |  | 250 | 60 | 60 | 100 |
|  |  | 125 | 60 | 60 | 100 |
|  |  | 62.5 | 60 | 60 | 100 |
|  | Prepared milk beverage C | 500 | 60 | 60 | 100 |
|  |  | 250 | 60 | 60 | 100 |
|  |  | 125 | 60 | 60 | 100 |
|  |  | 62.5 | 60 | 60 | 100 |
|  | Prepared milk drink D | 500 | 60 | 60 | 100 |
|  |  | 250 | 60 | 60 | 100 |
|  |  | 125 | 60 | 60 | 100 |
|  |  | 62.5 | 60 | 60 | 100 |

Note: survival rate=1-(N24-N0)/N0×100%
