# Supplementary material for: Effects of commercial beverages on the neurobehavioral motility of Caenorhabditis elegans
Source: PeerJ. 2022 Jul 14;10:e13563. doi: 10.7717/peerj.13563 (PMC9288823; doi:10.7717/peerj.13563)
Supplement: Supplemental Information 2 — Note: * is mean > control group and P < 0.05, # is mean < control group and P < 0.05. fold = treated group/control group. [file peerj-10-13563-s002.docx]

**Table S2 Effects of different types of beverages on the frequency of head thrash of nematodes**

| **category** | **sample** | **dose(μL/mL)** | **treated group** | | **control** | | **fold change** | **note** |
| --- | --- | --- | --- | --- | --- | --- | --- | --- |
|  |  |  | **mean** | SEM | **mean** | SEM |  |  |
| Fruit juice | mixed juice | 500 | 54.57 | 6.61 | 88.97 | 16.35 | 0.61 | # |
|  |  | 250 | 55.90 | 5.96 | 88.97 | 16.35 | 0.63 | # |
|  |  | 125 | 51.53 | 5.67 | 88.97 | 16.35 | 0.58 | # |
|  |  | 62.5 | 58.47 | 5.81 | 88.97 | 16.35 | 0.66 | # |
|  | Single juice | 500 | 89.47 | 14.62 | 88.97 | 16.35 | 1.01 |  |
|  |  | 250 | 88.57 | 11.55 | 88.97 | 16.35 | 1.00 |  |
|  |  | 125 | 66.40 | 7.23 | 88.97 | 16.35 | 0.75 | # |
|  |  | 62.5 | 66.20 | 7.80 | 88.97 | 16.35 | 0.74 | # |
| Carbonated drinks | Brown carbonated beverage | 500 | 60.80 | 10.50 | 79.60 | 15.15 | 0.76 | # |
|  |  | 250 | 55.33 | 6.44 | 79.60 | 15.15 | 0.70 | # |
|  |  | 125 | 60.93 | 9.06 | 79.60 | 15.15 | 0.77 | # |
|  |  | 62.5 | 54.97 | 10.71 | 79.60 | 15.15 | 0.69 | # |
|  | Colorless carbonated beverage | 500 | 69.93 | 19.65 | 79.60 | 15.15 | 0.88 | # |
|  |  | 250 | 83.23 | 16.30 | 79.60 | 15.15 | 1.05 |  |
|  |  | 125 | 59.33 | 4.43 | 79.60 | 15.15 | 0.75 | # |
|  |  | 62.5 | 53.60 | 6.49 | 79.60 | 15.15 | 0.67 | # |
|  | Orange carbonated beverage | 500 | 49.60 | 4.93 | 79.60 | 15.15 | 0.62 | # |
|  |  | 250 | 89.33 | 8.93 | 79.60 | 15.15 | 1.12 | * |
|  |  | 125 | 59.73 | 21.78 | 79.60 | 15.15 | 0.75 | # |
|  |  | 62.5 | 86.93 | 7.27 | 79.60 | 15.15 | 1.09 | * |
| Functional beverage | Sports functional drink | 500 | 89.6 | 19.53 | 60.07 | 7.27 | 1.49 | * |
|  |  | 250 | 82.00 | 17.16 | 60.07 | 7.27 | 1.37 | * |
|  |  | 125 | 67.87 | 21.55 | 60.07 | 7.27 | 1.13 | * |
|  |  | 62.5 | 59.80 | 7.83 | 60.07 | 7.27 | 1.00 |  |
|  | Fatigue relieving functional drink | 500 | 39.93 | 12.98 | 71.67 | 14.48 | 0.56 | # |
|  |  | 250 | 49.20 | 8.35 | 71.67 | 14.48 | 0.69 | # |
|  |  | 125 | 73.47 | 15.93 | 71.67 | 14.48 | 1.03 |  |
|  |  | 62.5 | 66.47 | 15.25 | 71.67 | 14.48 | 0.93 |  |

Note: * is mean > control group and *P* < 0.05, # is mean < control group and *P* < 0.05. fold=treated group/control group.

**Continued Table S1 Effects of different types of beverages on the frequency of**

**head thrash of nematodes**

| **category** | **sample** | **dose(μL/mL)** | **treated group** | | **control** | | **fold change** | **note** |
| --- | --- | --- | --- | --- | --- | --- | --- | --- |
|  |  |  | mean | SEM | mean | SEM |  |  |
| Tea beverage | Black tea beverage | 500 | 91.67 | 8.17 | 60.07 | 7.27 | 1.53 | * |
|  |  | 250 | 89.67 | 8.10 | 60.07 | 7.27 | 1.49 | * |
|  |  | 125 | 42.50 | 10.49 | 60.07 | 7.27 | 0.71 | # |
|  |  | 62.5 | 45.93 | 6.24 | 60.07 | 7.27 | 0.76 | # |
|  | Green tea beverage | 500 | 53.17 | 8.21 | 49.07 | 4.46 | 1.08 |  |
|  |  | 250 | 53.43 | 6.66 | 49.07 | 4.46 | 1.09 | * |
|  |  | 125 | 60.53 | 7.01 | 49.07 | 4.46 | 1.23 | * |
|  |  | 62.5 | 59.90 | 6.51 | 49.07 | 4.46 | 1.22 | * |
|  | Herbal tea drink | 500 | 40.30 | 4.41 | 49.07 | 4.46 | 0.82 | # |
|  |  | 250 | 69.87 | 16.39 | 49.07 | 4.46 | 1.42 | * |
|  |  | 125 | 43.03 | 3.90 | 49.07 | 4.46 | 0.88 | # |
|  |  | 62.5 | 68.57 | 10.95 | 49.07 | 4.46 | 1.40 | * |
| Coffee beverage | Coffee drinks | 500 | 45.53 | 11.22 | 49.07 | 4.46 | 0.93 |  |
|  |  | 250 | 49.73 | 4.91 | 49.07 | 4.46 | 1.01 |  |
|  |  | 125 | 46.93 | 6.74 | 49.07 | 4.46 | 0.96 |  |
|  |  | 62.5 | 46.80 | 6.27 | 49.07 | 4.46 | 0.95 |  |
| Phytoprotein beverage | Almond milk | 500 | 46.13 | 10.60 | 46.00 | 8.94 | 1.00 |  |
|  |  | 250 | 45.20 | 8.36 | 46.00 | 8.94 | 0.98 |  |
|  |  | 125 | 49.37 | 8.99 | 46.00 | 8.94 | 1.07 |  |
|  |  | 62.5 | 45.47 | 12.97 | 46.00 | 8.94 | 0.99 |  |
|  | Coconut drink | 500 | 33.93 | 5.60 | 46.00 | 8.94 | 0.74 | # |
|  |  | 250 | 40.00 | 5.04 | 46.00 | 8.94 | 0.87 | # |
|  |  | 125 | 42.93 | 4.83 | 46.00 | 8.94 | 0.93 |  |
|  |  | 62.5 | 57.00 | 11.84 | 46.00 | 8.94 | 1.24 | * |
|  | Milk tea beverage | 500 | 54.63 | 3.64 | 46.00 | 8.94 | 1.19 | * |
|  |  | 250 | 61.87 | 6.56 | 46.00 | 8.94 | 1.35 | * |
|  |  | 125 | 50.63 | 6.59 | 46.00 | 8.94 | 1.10 | * |
|  |  | 62.5 | 49.93 | 6.05 | 46.00 | 8.94 | 1.09 |  |

Note: * is mean > control group and *P* < 0.05, # is mean < control group and *P* < 0.05. fold=treated group/control group.

**Continued Table S1 Effects of different types of beverages on the frequency of**

**head thrash of nematodes**

| **category** | **sample** | **dose(μL/mL)** | **treated group** | | **control** | | **fold change** | **note** |
| --- | --- | --- | --- | --- | --- | --- | --- | --- |
|  |  |  | mean | SEM | mean | SEM |  |  |
| Dairy products | Prepared milk beverage A | 500 | 80.47 | 16.68 | 58.60 | 5.04 | 1.37 | * |
|  |  | 250 | 87.73 | 8.23 | 58.60 | 5.04 | 1.50 | * |
|  |  | 125 | 91.97 | 9.27 | 58.60 | 5.04 | 1.57 | * |
|  |  | 62.5 | 90.93 | 9.54 | 58.60 | 5.04 | 1.55 | * |
|  | Prepared milk beverage B | 500 | 80.73 | 13.14 | 58.60 | 5.04 | 1.38 | * |
|  |  | 250 | 87.20 | 10.03 | 58.60 | 5.04 | 1.49 | * |
|  |  | 125 | 48.67 | 5.99 | 58.60 | 5.04 | 0.83 | # |
|  |  | 62.5 | 46.70 | 6.36 | 58.60 | 5.04 | 0.80 | # |
|  | Prepared milk beverage C | 500 | 56.27 | 5.67 | 71.67 | 14.48 | 0.79 | # |
|  |  | 250 | 57.53 | 6.64 | 71.67 | 14.48 | 0.80 | # |
|  |  | 125 | 59.47 | 6.24 | 71.67 | 14.48 | 0.83 | # |
|  |  | 62.5 | 64.27 | 8.48 | 71.67 | 14.48 | 0.90 | # |
|  | Prepared milk drink D | 500 | 55.43 | 7.88 | 71.67 | 14.48 | 0.77 | # |
|  |  | 250 | 76.07 | 13.72 | 71.67 | 14.48 | 1.06 |  |
|  |  | 125 | 58.27 | 7.23 | 71.67 | 14.48 | 0.81 | # |
|  |  | 62.5 | 62.53 | 12.51 | 71.67 | 14.48 | 0.87 | # |

Note: * is mean > control group and *P* < 0.05, # is mean < control group and *P* < 0.05. fold=treated group/control group.
