# Supplementary material for: Effects of commercial beverages on the neurobehavioral motility of Caenorhabditis elegans
Source: PeerJ. 2022 Jul 14;10:e13563. doi: 10.7717/peerj.13563 (PMC9288823; doi:10.7717/peerj.13563)
Supplement: Supplemental Information 3 — Note: * is mean > control group and P < 0.05, # is mean < control group and P < 0.05. fold = treated group/control group. [file peerj-10-13563-s003.docx]

**Table S3 Effects of different types of beverages on the frequency of body bend of nematodes**

| **category** | **sample** | **dose(μL/mL)** | **treated group** | | **control** | | **fold change** | **note** |
| --- | --- | --- | --- | --- | --- | --- | --- | --- |
|  |  |  | mean | SEM | mean | SEM |  |  |
| Fruit juice | mixed juice | 500 | 6.70 | 2.38 | 8.97 | 1.54 | 0.75 | # |
|  |  | 250 | 3.77 | 1.01 | 8.97 | 1.54 | 0.42 | # |
|  |  | 125 | 4.23 | 1.10 | 8.97 | 1.54 | 0.47 | # |
|  |  | 62.5 | 3.57 | 1.04 | 8.97 | 1.54 | 0.40 | # |
|  | Single juice | 500 | 9.17 | 2.53 | 8.97 | 1.54 | 1.02 |  |
|  |  | 250 | 9.10 | 2.32 | 8.97 | 1.54 | 1.01 |  |
|  |  | 125 | 6.93 | 1.80 | 8.97 | 1.54 | 0.77 | # |
|  |  | 62.5 | 7.70 | 1.82 | 8.97 | 1.54 | 0.86 | # |
| Carbonated drinks | Brown carbonated beverage | 500 | 5.17 | 1.18 | 6.17 | 1.90 | 0.84 | # |
|  |  | 250 | 5.20 | 1.21 | 6.17 | 1.90 | 0.84 | # |
|  |  | 125 | 5.07 | 1.55 | 6.17 | 1.90 | 0.82 | # |
|  |  | 62.5 | 4.60 | 1.38 | 6.17 | 1.90 | 0.75 | # |
|  | Colorless carbonated beverage | 500 | 4.83 | 1.97 | 6.17 | 1.90 | 0.78 | # |
|  |  | 250 | 5.47 | 1.81 | 6.17 | 1.90 | 0.89 |  |
|  |  | 125 | 4.70 | 1.66 | 6.17 | 1.90 | 0.76 | # |
|  |  | 62.5 | 3.80 | 1.40 | 6.17 | 1.90 | 0.62 | # |
|  | Orange carbonated beverage | 500 | 4.70 | 1.90 | 6.17 | 1.90 | 0.76 | # |
|  |  | 250 | 8.13 | 1.17 | 6.17 | 1.90 | 1.32 | * |
|  |  | 125 | 6.17 | 1.86 | 6.17 | 1.90 | 1.00 |  |
|  |  | 62.5 | 8.13 | 1.70 | 6.17 | 1.90 | 1.32 | * |
| Functional beverage | Sports functional drink | 500 | 6.17 | 1.97 | 4.53 | 1.33 | 1.36 | * |
|  |  | 250 | 6.43 | 2.05 | 4.53 | 1.33 | 1.42 | * |
|  |  | 125 | 6.50 | 2.69 | 4.53 | 1.33 | 1.43 | * |
|  |  | 62.5 | 5.10 | 1.45 | 4.53 | 1.33 | 1.13 | * |
|  | Fatigue relieving functional drink | 500 | 2.03 | 1.03 | 6.70 | 1.47 | 0.30 | # |
|  |  | 250 | 4.13 | 1.83 | 6.70 | 1.47 | 0.62 | # |
|  |  | 125 | 5.73 | 1.57 | 6.70 | 1.47 | 0.86 | # |
|  |  | 62.5 | 5.37 | 1.81 | 6.70 | 1.47 | 0.80 | # |

Note: * is mean > control group and *P* < 0.05, # is mean < control group and *P* < 0.05. fold=treated group/control group.

**Continued Table S2 Effects of different types of beverages on the frequency of**

**body bend of nematodes**

| **category** | **sample** | **dose(μL/mL)** | **treated group** | | **control** | | **fold change** | **note** |
| --- | --- | --- | --- | --- | --- | --- | --- | --- |
|  |  |  | mean | SEM | mean | SEM |  |  |
| Tea beverage | Black tea beverage | 500 | 7.20 | 1.90 | 4.53 | 1.33 | 1.59 | * |
|  |  | 250 | 8.47 | 2.10 | 4.53 | 1.33 | 1.87 |  |
|  |  | 125 | 3.90 | 1.16 | 4.53 | 1.33 | 0.86 |  |
|  |  | 62.5 | 4.07 | 1.70 | 4.53 | 1.33 | 0.90 |  |
|  | Green tea flavored beverage | 500 | 5.07 | 1.84 | 6.53 | 1.36 | 0.78 | # |
|  |  | 250 | 5.53 | 1.89 | 6.53 | 1.36 | 0.85 | # |
|  |  | 125 | 4.63 | 1.81 | 6.53 | 1.36 | 0.71 |  |
|  |  | 62.5 | 5.70 | 1.73 | 6.53 | 1.36 | 0.87 | # |
|  | Herbal tea drink | 500 | 7.33 | 1.63 | 6.53 | 1.36 | 1.12 |  |
|  |  | 250 | 7.53 | 1.25 | 6.53 | 1.36 | 1.15 | * |
|  |  | 125 | 6.67 | 2.02 | 6.53 | 1.36 | 1.02 |  |
|  |  | 62.5 | 7.47 | 1.63 | 6.53 | 1.36 | 1.14 | * |
| Coffee beverage | Coffee | 500 | 5.10 | 1.97 | 6.53 | 1.36 | 0.78 | # |
|  |  | 250 | 5.70 | 1.51 | 6.53 | 1.36 | 0.87 |  |
|  |  | 125 | 5.60 | 1.48 | 6.53 | 1.36 | 0.86 | # |
|  |  | 62.5 | 5.17 | 1.51 | 6.53 | 1.36 | 0.79 | # |
| Phytoprotein beverage | Almond milk | 500 | 8.40 | 2.50 | 5.57 | 1.59 | 1.51 | * |
|  |  | 250 | 9.80 | 3.52 | 5.57 | 1.59 | 1.76 | * |
|  |  | 125 | 7.93 | 1.72 | 5.57 | 1.59 | 1.42 | * |
|  |  | 62.5 | 9.83 | 3.44 | 5.57 | 1.59 | 1.76 | * |
|  | Coconut drink | 500 | 6.21 | 1.23 | 5.57 | 1.59 | 1.11 |  |
|  |  | 250 | 8.40 | 2.51 | 5.57 | 1.59 | 1.51 | * |
|  |  | 125 | 10.83 | 3.13 | 5.57 | 1.59 | 1.94 | * |
|  |  | 62.5 | 8.00 | 2.18 | 5.57 | 1.59 | 1.44 | * |
|  | Milk tea beverage | 500 | 6.40 | 2.42 | 5.57 | 1.59 | 1.15 |  |
|  |  | 250 | 6.97 | 2.83 | 5.57 | 1.59 | 1.25 | * |
|  |  | 125 | 5.80 | 1.63 | 5.57 | 1.59 | 1.04 |  |
|  |  | 62.5 | 6.10 | 1.52 | 5.57 | 1.59 | 1.10 |  |

Note: * is mean > control group and *P* < 0.05, # is mean < control group and *P* < 0.05. fold=treated group/control group.

**Continued Table S2 Effects of different types of beverages on the frequency of**

**body bend of nematodes**

| **category** | **sample** | **dose(μL/mL)** | **treated group** | | **control** | | **fold change** | **note** |
| --- | --- | --- | --- | --- | --- | --- | --- | --- |
|  |  |  | mean | SEM | mean | SEM |  |  |
| Dairy products | Prepared milk beverage A | 500 | 8.17 | 1.51 | 5.47 | 0.86 | 1.49 | * |
|  |  | 250 | 10.23 | 1.65 | 5.47 | 0.86 | 1.87 | * |
|  |  | 125 | 10.47 | 1.83 | 5.47 | 0.86 | 1.91 | * |
|  |  | 62.5 | 9.93 | 1.39 | 5.47 | 0.86 | 1.82 | * |
|  | Prepared milk beverage B | 500 | 9.10 | 1.58 | 5.47 | 0.86 | 1.66 | * |
|  |  | 250 | 10.00 | 2.32 | 5.47 | 0.86 | 1.83 | * |
|  |  | 125 | 5.47 | 1.22 | 5.47 | 0.86 | 1.00 |  |
|  |  | 62.5 | 5.17 | 1.34 | 5.47 | 0.86 | 0.95 |  |
|  | Prepared milk beverage C | 500 | 5.03 | 1.56 | 6.70 | 1.47 | 0.75 | # |
|  |  | 250 | 5.33 | 1.09 | 6.70 | 1.47 | 0.80 | # |
|  |  | 125 | 5.83 | 1.64 | 6.70 | 1.47 | 0.87 | # |
|  |  | 62.5 | 6.17 | 1.32 | 6.70 | 1.47 | 0.92 |  |
|  | Prepared milk drink D | 500 | 5.93 | 1.93 | 6.70 | 1.47 | 0.89 |  |
|  |  | 250 | 9.60 | 1.75 | 6.70 | 1.47 | 1.43 | * |
|  |  | 125 | 7.50 | 2.08 | 6.70 | 1.47 | 1.12 |  |
|  |  | 62.5 | 7.20 | 1.88 | 6.70 | 1.47 | 1.07 |  |

Note: * is mean > control group and *P* < 0.05, # is mean < control group and *P* < 0.05. fold=treated group/control group.
