# Supplementary material for: Effects of commercial beverages on the neurobehavioral motility of Caenorhabditis elegans
Source: PeerJ. 2022 Jul 14;10:e13563. doi: 10.7717/peerj.13563 (PMC9288823; doi:10.7717/peerj.13563)
Supplement: Supplemental Information 4 — Note: * is mean > control group and P < 0.05, # is mean < control group and P < 0.05. fold = treated group/control group. [file peerj-10-13563-s004.docx]

**Table S4 Effects of different types of beverages on the frequency of pharyngeal pump of nematodes**

| **category** | **sample** | **dose(μL/mL)** | **treated group** | | **control** | | **fold change** | **note** |
| --- | --- | --- | --- | --- | --- | --- | --- | --- |
|  |  |  | mean | SEM | mean | SEM |  |  |
| Fruit juice | mixed juice | 500 | 35.45 | 18.34 | 61.35 | 8.88 | 0.58 | # |
|  |  | 250 | 52.50 | 12.03 | 61.35 | 8.88 | 0.86 | # |
|  |  | 125 | 56.05 | 7.97 | 61.35 | 8.88 | 0.91 |  |
|  |  | 62.5 | 48.65 | 15.18 | 61.35 | 8.88 | 0.79 | # |
|  | Single juice | 500 | 45.90 | 17.63 | 61.35 | 8.88 | 0.75 | # |
|  |  | 250 | 54.10 | 10.16 | 61.35 | 8.88 | 0.88 |  |
|  |  | 125 | 56.68 | 10.05 | 61.35 | 8.88 | 0.92 |  |
|  |  | 62.5 | 59.00 | 8.16 | 61.35 | 8.88 | 0.96 |  |
| Carbonated drinks | Brown carbonated beverage | 500 | 59.37 | 10.06 | 65.50 | 6.92 | 0.91 |  |
|  |  | 250 | 60.37 | 5.78 | 65.50 | 6.92 | 0.92 |  |
|  |  | 125 | 56.74 | 12.06 | 65.50 | 6.92 | 0.87 | # |
|  |  | 62.5 | 54.65 | 15.64 | 65.50 | 6.92 | 0.83 | # |
|  | Colorless carbonated beverage | 500 | 59.89 | 9.06 | 65.50 | 6.92 | 0.91 |  |
|  |  | 250 | 56.75 | 5.67 | 65.50 | 6.92 | 0.87 | # |
|  |  | 125 | 58.05 | 10.99 | 65.50 | 6.92 | 0.89 | # |
|  |  | 62.5 | 62.45 | 8.13 | 65.50 | 6.92 | 0.95 |  |
|  | Orange carbonated beverage | 500 | 56.10 | 8.63 | 65.50 | 6.92 | 0.86 | # |
|  |  | 250 | 58.95 | 8.43 | 65.50 | 6.92 | 0.90 | # |
|  |  | 125 | 59.74 | 9.94 | 65.50 | 6.92 | 0.91 |  |
|  |  | 62.5 | 56.20 | 12.86 | 65.50 | 6.92 | 0.86 | # |
| Functional beverage | Sports functional drink | 500 | 56.89 | 5.73 | 52.79 | 6.29 | 1.08 |  |
|  |  | 250 | 51.35 | 9.20 | 52.79 | 6.29 | 0.97 |  |
|  |  | 125 | 51.61 | 8.96 | 52.79 | 6.29 | 0.98 |  |
|  |  | 62.5 | 52.40 | 7.30 | 52.79 | 6.29 | 0.99 |  |
|  | Fatigue relieving functional drink | 500 | 37.78 | 4.95 | 53.60 | 20.00 | 0.70 | # |
|  |  | 250 | 42.71 | 6.02 | 53.60 | 20.00 | 0.80 | # |
|  |  | 125 | 43.30 | 5.76 | 53.60 | 20.00 | 0.81 | # |
|  |  | 62.5 | 39.20 | 4.95 | 53.60 | 20.00 | 0.73 | # |

Note: * is mean > control group and *P* < 0.05, # is mean < control group and *P* < 0.05. fold=treated group/control group.

**Continued Table S3 Effects of different types of beverages on the frequency of**

**pharyngeal pump of nematodes**

| **category** | **sample** | **dose(μL/mL)** | **treated group** | | **control** | | **fold change** | **note** |
| --- | --- | --- | --- | --- | --- | --- | --- | --- |
|  |  |  | mean | SEM | mean | SEM |  |  |
| Tea beverage | Black tea beverage | 500 | 34.65 | 11.55 | 52.79 | 6.29 | 0.66 | # |
|  |  | 250 | 46.43 | 6.28 | 52.79 | 6.29 | 0.88 | # |
|  |  | 125 | 47.60 | 5.70 | 52.79 | 6.29 | 0.90 | # |
|  |  | 62.5 | 49.35 | 5.20 | 52.79 | 6.29 | 0.93 |  |
|  | Green tea beverage | 500 | 47.68 | 8.79 | 55.6 | 7.70 | 0.86 | # |
|  |  | 250 | 53.90 | 5.61 | 55.6 | 7.70 | 0.97 |  |
|  |  | 125 | 49.60 | 9.72 | 55.6 | 7.70 | 0.89 | # |
|  |  | 62.5 | 48.40 | 9.19 | 55.6 | 7.70 | 0.87 | # |
|  | Herbal tea drink | 500 | 46.80 | 7.25 | 55.6 | 7.70 | 0.84 | # |
|  |  | 250 | 49.25 | 9.54 | 55.6 | 7.70 | 0.89 | # |
|  |  | 125 | 49.30 | 8.14 | 55.6 | 7.70 | 0.89 | # |
|  |  | 62.5 | 53.20 | 11.86 | 55.6 | 7.70 | 0.96 |  |
| Coffee beverage | Coffee drinks | 500 | 56.15 | 6.93 | 55.6 | 7.70 | 1.01 |  |
|  |  | 250 | 47.80 | 7.43 | 55.6 | 7.70 | 0.86 | # |
|  |  | 125 | 45.35 | 8.18 | 55.6 | 7.70 | 0.82 | # |
|  |  | 62.5 | 50.45 | 9.25 | 55.6 | 7.70 | 0.91 |  |
| Phytoprotein beverage | Almond milk | 500 | 51.80 | 14.29 | 41.4 | 7.18 | 1.25 | * |
|  |  | 250 | 52.00 | 7.24 | 41.4 | 7.18 | 1.26 | * |
|  |  | 125 | 47.18 | 9.59 | 41.4 | 7.18 | 1.14 | * |
|  |  | 62.5 | 46.68 | 11.15 | 41.4 | 7.18 | 1.13 |  |
|  | Coconut drink | 500 | 46.10 | 9.59 | 41.4 | 7.18 | 1.11 |  |
|  |  | 250 | 46.81 | 8.25 | 41.4 | 7.18 | 1.13 |  |
|  |  | 125 | 48.26 | 7.92 | 41.4 | 7.18 | 1.17 | * |
|  |  | 62.5 | 54.05 | 7.31 | 41.4 | 7.18 | 1.31 | * |
|  | Milk tea beverage | 500 | 48.70 | 7.80 | 41.4 | 7.18 | 1.18 | * |
|  |  | 250 | 52.50 | 7.29 | 41.4 | 7.18 | 1.27 | * |
|  |  | 125 | 60.30 | 7.89 | 41.4 | 7.18 | 1.46 | * |
|  |  | 62.5 | 57.85 | 9.18 | 41.4 | 7.18 | 1.40 | * |

Note: * is mean > control group and *P* < 0.05, # is mean < control group and *P* < 0.05. fold=treated group/control group.

**Continued Table S3 Effects of different types of beverages on the frequency of**

**pharyngeal pump of nematodes**

| **category** | **sample** | **dose(μL/mL)** | **treated group** | | **control** | | **fold change** | **note** |
| --- | --- | --- | --- | --- | --- | --- | --- | --- |
|  |  |  | mean | SEM | mean | SEM |  |  |
| Dairy products | Prepared milk beverage A | 500 | 53.74 | 6.38 | 50.7 | 9.81 | 1.06 |  |
|  |  | 250 | 55.75 | 6.45 | 50.7 | 9.81 | 1.10 | * |
|  |  | 125 | 52.35 | 8.25 | 50.7 | 9.81 | 1.03 |  |
|  |  | 62.5 | 56.7 | 7.78 | 50.7 | 9.81 | 1.12 | * |
|  | Prepared milk beverage B | 500 | 57.9 | 8.83 | 50.7 | 9.81 | 1.14 | * |
|  |  | 250 | 53.5 | 6.07 | 50.7 | 9.81 | 1.06 |  |
|  |  | 125 | 56.4 | 8.02 | 50.7 | 9.81 | 1.11 | * |
|  |  | 62.5 | 59.75 | 6.34 | 50.7 | 9.81 | 1.18 | * |
|  | Prepared milk beverage C | 500 | 49.95 | 5.04 | 53.6 | 20 | 0.93 |  |
|  |  | 250 | 53.65 | 7.19 | 53.6 | 20 | 1.00 |  |
|  |  | 125 | 48.11 | 3.72 | 53.6 | 20 | 0.90 |  |
|  |  | 62.5 | 57.39 | 8.18 | 53.6 | 20 | 1.07 |  |
|  | Prepared milk drink D | 500 | 48.84 | 11.16 | 53.6 | 20 | 0.91 |  |
|  |  | 250 | 47.6 | 7.89 | 53.6 | 20 | 0.89 | # |
|  |  | 125 | 43.84 | 9.28 | 53.6 | 20 | 0.82 | # |
|  |  | 62.5 | 40.9 | 12.69 | 53.6 | 20 | 0.76 | # |

Note: * is mean > control group and *P* < 0.05, # is mean < control group and *P* < 0.05. fold=treated group/control group.
