# Supplementary material for: Effects of commercial beverages on the neurobehavioral motility of Caenorhabditis elegans
Source: PeerJ. 2022 Jul 14;10:e13563. doi: 10.7717/peerj.13563 (PMC9288823; doi:10.7717/peerj.13563)
Supplement: Supplemental Information 5 [file peerj-10-13563-s005.docx]

**Table S5 Chemical compositions of beverages**

| **category** | **sample** | **chemical compositions** |
| --- | --- | --- |
| Fruit juice | mixed juice | 100% apple juice, 100% banana puree |
|  | Single juice | 100% orange juice, orange pulp (5% addition) |
| Carbonated drinks | Brown carbonated beverage | water, fructose syrup, sugar, food additives (carbon dioxide, caramel, phosphoric acid, caffeine), edible essence |
|  | Colorless carbonated beverage | water, fruit syrup, white granulated sugar, food additives (carbon dioxide, lemon yellow, sodium citrate, sodium benzoate), edible essence |
|  | Orange carbonated beverage | water, fructose syrup, white granulated sugar, maltodextrin, food additives (carbon dioxide, citric acid, hexametaphosphoric acid, potassium sorbate, edible essence, acsyl, vitamin C, sucrose, sunset yellow, lemon yellow, sodium citrate, carmine) |
| Functional beverage | Sports functional drink | water, white granulated sugar, apple juice, edible essence, food additives (citric acid, sodium citrate), vitamin C, nicotinamide, vitamin B6, vitamin B12 |
|  | Fatigue relieving functional drink | each 100ml contains 190mg taurine, 20mg caffeine, 9.0mg inositol, 4.0mg nicotinamide, 1.7mg pantothenic acid, 3.2mg total saponins and 0.4mg vitamin B6 |
| Tea beverage | Black tea beverage | water, white granulated sugar, edible salt, instant black tea, food additives (citric acid, sodium citrate, vitamin C, caramel), edible essence |
|  | Green tea beverage | water, white granulated sugar, Jasmine tea leaves (green tea billet), green tea leaves, honey (0.3g/kg), concentrated green tea solution, food additives (D-isoascorbate sodium, hexametaphosphoric acid, sodium citrate, vitamin C, sodium bicarbonate), edible essence |
|  | Herbal tea drink | water, white granulated sugar, Mesona chinensis Benth, Plumeria rubra 'Acutifolia', Microcos paniculata Linn, Chrysanthemum × morifolium Ramat, Lonicera japonica Thunb, Prunella vulgaris L, Glycyrrhiza uralensis Fisch |
| Coffee beverage | Coffee drinks | water, milk powder (≥6%), instant coffee (≥0.9%), food additives (microcrystalline cellulose, sodium bicarbonate sucrose fatty acid ester, sodium carboxymethyl cellulose, sodium citrate), food essence |

**Continued Table S4 Chemical compositions of beverages**

| **category** | **sample** | **chemical compositions** |
| --- | --- | --- |
| Phytoprotein beverage | Almond milk | water, white granulated sugar, polyglycerol fatty acid ester, sodium bicarbonate, citric acid, food spices |
|  | Coconut drink | water, fresh coconut juice, white granulated sugar, food additives (sodium caseinate, glyceryl monostearate) |
|  | Milk tea beverage | water, white granulated sugar, glucose syrup, milk powder, coconut oil, black tea, emulsifier (sodium caseinate), acidity regulator, edible essence, antioxidants. |
| Dairy products | Prepared milk beverage A | water, raw milk, white granulated sugar, whole milk powder, whey protein, fructose syrup, concentrated apple juice, food additives (sodium carboxymethyl cellulose, xanthan gum, lactic acid, citric acid, sodium citrate, single or double glycerin fatty acid ester, propylene glycol alginate, succinic acid monoglyceride, diacetyl tartaric acid and diglycerides, sucralose, arcelormittal honey), new sweet, food in essence |
|  | Prepared milk beverage B | water, white granulated sugar, milk powder (whole milk powder, skim milk powder), food additives (carboxymethyl cellulose sodium, citric acid, sodium tripolyphosphate, hydroxypropyl two starch phosphate guar gum, aspartame, acsam, monostearic acid glyceride, sucrose fatty acid ester, lactostreptococcus), whey protein concentrate, edible essence, taurine, zinc citrate, nicotinamide, Streptococcus thermophilus, Lactobacillus bulgaricus |
|  | Prepared milk beverage C | water, white granulated sugar, whole milk powder, food additives, concentrated apple juice, skimmed milk powder, concentrated whey protein, edible essence, taurine, zinc citrate, niacinamide, probiotics (Streptococcus thermophilus, Lactobacillus bulgaricus) |
|  | Prepared milk drink D | water, white granulated sugar, whole milk powder, food additives (carboxymethyl cellulose, sodium phosphate, citric acid, sodium tripolyphosphate, sodium citrate, glycerol monostearate, sucrose fatty acid ester, potassium sorbate, aspartame, acaside, nisin), whey protein concentrate, calcium carbonate, edible essence, vitamin A, vitamin D |
