# Supplementary material for: Effects of commercial beverages on the neurobehavioral motility of Caenorhabditis elegans
Source: PeerJ. 2022 Jul 14;10:e13563. doi: 10.7717/peerj.13563 (PMC9288823; doi:10.7717/peerj.13563)
Supplement: Supplemental Information 6 [file peerj-10-13563-s006.docx]

**Table S6--raw data--Neurobehavioral changes of nematodes treated by mixed juice**

| **No.** | **body bend** | | | | | **head thrash** | | | | | **pharyngeal pump** | | | | |
| --- | --- | --- | --- | --- | --- | --- | --- | --- | --- | --- | --- | --- | --- | --- | --- |
|  | 500 | 250 | 125 | 62.5 | ctr | 500 | 250 | 125 | 62.5 | ctr | 500 | 250 | 125 | 62.5 | ctr |
| 1 | 7 | 4 | 8 | 5 | 11 | 72 | 49 | 44 | 67 | 88 | 44 | 30 | 67 | 58 | 70 |
| 2 | 9 | 5 | 4 | 4 | 10 | 69 | 64 | 48 | 63 | 106 | 14 | 58 | 64 | 65 | 69 |
| 3 | 6 | 3 | 4 | 6 | 9 | 56 | 61 | 50 | 63 | 72 | 36 | 46 | 62 | 59 | 65 |
| 4 | 11 | 5 | 3 | 5 | 11 | 62 | 62 | 46 | 60 | 50 | 17 | 54 | 67 | 47 | 64 |
| 5 | 9 | 5 | 4 | 4 | 10 | 58 | 57 | 44 | 56 | 88 | 39 | 53 | 58 | 66 | 69 |
| 6 | 12 | 3 | 3 | 3 | 5 | 61 | 58 | 54 | 59 | 92 | 13 | 45 | 61 | 48 | 71 |
| 7 | 10 | 4 | 4 | 4 | 9 | 51 | 53 | 48 | 60 | 82 | 11 | 72 | 53 | 60 | 68 |
| 8 | 9 | 6 | 2 | 5 | 10 | 53 | 62 | 50 | 66 | 102 | 51 | 63 | 46 | 57 | 70 |
| 9 | 10 | 3 | 5 | 3 | 11 | 55 | 49 | 54 | 67 | 76 | 40 | 10 | 60 | 48 | 66 |
| 10 | 6 | 3 | 5 | 2 | 10 | 51 | 57 | 58 | 57 | 62 | 53 | 73 | 63 | 39 | 48 |
| 11 | 7 | 4 | 6 | 5 | 9 | 48 | 66 | 40 | 47 | 102 | 44 | 68 | 44 | 48 | 69 |
| 12 | 9 | 5 | 4 | 3 | 7 | 51 | 68 | 48 | 60 | 112 | 57 | 55 | 62 | 56 | 37 |
| 13 | 4 | 5 | 5 | 3 | 9 | 48 | 61 | 42 | 49 | 74 | 23 | 57 | 51 | 34 | 56 |
| 14 | 6 | 3 | 5 | 4 | 9 | 49 | 53 | 52 | 55 | 100 | 54 | 50 | 51 | 62 | 51 |
| 15 | 5 | 4 | 4 | 3 | 11 | 56 | 57 | 54 | 53 | 62 | 14 | 54 | 42 | 63 | 23 |
| 16 | 5 | 4 | 4 | 4 | 10 | 53 | 56 | 56 | 48 | 90 | 58 | 33 | 64 | 65 | 62 |
| 17 | 4 | 3 | 3 | 3 | 9 | 50 | 49 | 58 | 57 | 98 | 17 | 57 | 46 | 26 | 61 |
| 18 | 6 | 4 | 4 | 3 | 10 | 58 | 54 | 60 | 48 | 102 | 11 | 36 | 54 | 30 | 59 |
| 19 | 5 | 3 | 5 | 2 | 8 | 50 | 50 | 58 | 53 | 100 | 55 | 53 | 58 | 23 | 61 |
| 20 | 4 | 5 | 4 | 4 | 7 | 47 | 52 | 52 | 57 | 72 | 58 | 56 | 48 | 19 | 58 |
| 21 | 5 | 3 | 4 | 3 | 10 | 65 | 50 | 60 | 59 | 92 | 37 |  |  |  | 53 |
| 22 | 5 | 4 | 4 | 4 | 8 | 63 | 52 | 44 | 69 | 94 |  |  |  |  |  |
| 23 | 9 | 3 | 5 | 5 | 9 | 47 | 56 | 58 | 61 | 80 |  |  |  |  |  |
| 24 | 4 | 3 | 4 | 3 | 7 | 46 | 53 | 52 | 63 | 98 |  |  |  |  |  |
| 25 | 7 | 4 | 3 | 2 | 10 | 56 | 58 | 56 | 57 | 88 |  |  |  |  |  |
| 26 | 6 | 5 | 4 | 3 | 9 | 53 | 57 | 48 | 58 | 122 |  |  |  |  |  |
| 27 | 3 | 2 | 5 | 4 | 7 | 52 | 61 | 52 | 62 | 108 |  |  |  |  |  |
| 28 | 6 | 3 | 4 | 2 | 6 | 48 | 44 | 60 | 56 | 92 |  |  |  |  |  |
| 29 | 4 | 3 | 3 | 3 | 10 | 53 | 62 | 48 | 59 | 76 |  |  |  |  |  |
| 30 | 8 | 2 | 5 | 3 | 8 | 56 | 46 | 52 | 65 |  |  |  |  |  |  |

Note: ctrl means *control group*; the unit of dose is *μL/mL*
