# Supplementary material for: Effects of commercial beverages on the neurobehavioral motility of Caenorhabditis elegans
Source: PeerJ. 2022 Jul 14;10:e13563. doi: 10.7717/peerj.13563 (PMC9288823; doi:10.7717/peerj.13563)
Supplement: Supplemental Information 7 — raw data [file peerj-10-13563-s007.docx]

**Table S7--raw data--Neurobehavioral changes of nematodes treated by single juice**

| **No.** | **body bend** | | | | | **head thrash** | | | | | **pharyngeal pump** | | | | |
| --- | --- | --- | --- | --- | --- | --- | --- | --- | --- | --- | --- | --- | --- | --- | --- |
|  | 500 | 250 | 125 | 62.5 | ctr | 500 | 250 | 125 | 62.5 | ctr | 500 | 250 | 125 | 62.5 | ctr |
| 1 | 8 | 13 | 11 | 10 | 11 | 94 | 80 | 74 | 78 | 88 | 24 | 48 | 61 | 53 | 70 |
| 2 | 4 | 8 | 5 | 7 | 10 | 102 | 90 | 60 | 70 | 106 | 2 | 52 | 56 | 37 | 69 |
| 3 | 8 | 9 | 7 | 8 | 9 | 86 | 88 | 58 | 68 | 72 | 48 | 54 | 71 | 49 | 65 |
| 4 | 5 | 11 | 4 | 6 | 11 | 83 | 86 | 72 | 62 | 50 | 30 | 38 | 61 | 68 | 64 |
| 5 | 13 | 7 | 10 | 7 | 10 | 60 | 90 | 76 | 62 | 88 | 54 | 65 | 71 | 54 | 69 |
| 6 | 7 | 11 | 5 | 7 | 5 | 80 | 84 | 72 | 68 | 92 | 68 | 61 | 46 | 60 | 71 |
| 7 | 11 | 12 | 7 | 5 | 9 | 81 | 101 | 78 | 62 | 82 | 59 | 57 | 44 | 62 | 68 |
| 8 | 10 | 9 | 6 | 6 | 10 | 82 | 78 | 68 | 74 | 102 | 35 | 68 | 65 | 54 | 70 |
| 9 | 11 | 10 | 10 | 6 | 11 | 54 | 98 | 78 | 64 | 76 | 40 | 60 | 52 | 63 | 66 |
| 10 | 5 | 6 | 5 | 10 | 10 | 101 | 81 | 68 | 58 | 62 | 70 | 58 | 47 | 61 | 48 |
| 11 | 10 | 8 | 6 | 9 | 9 | 96 | 104 | 72 | 54 | 102 | 62 | 62 | 69 | 72 | 69 |
| 12 | 9 | 5 | 10 | 6 | 7 | 90 | 96 | 68 | 84 | 112 | 61 | 52 | 59 | 63 | 37 |
| 13 | 9 | 8 | 9 | 8 | 9 | 98 | 106 | 60 | 82 | 74 | 26 | 39 | 50 | 44 | 56 |
| 14 | 10 | 9 | 6 | 5 | 9 | 69 | 74 | 58 | 66 | 100 | 55 | 56 | 55 | 63 | 51 |
| 15 | 12 | 8 | 7 | 7 | 11 | 80 | 92 | 70 | 64 | 62 | 68 | 34 | 40 | 75 | 23 |
| 16 | 11 | 13 | 7 | 7 | 10 | 83 | 104 | 58 | 68 | 90 | 39 | 48 | 58 | 64 | 62 |
| 17 | 13 | 5 | 5 | 9 | 9 | 100 | 86 | 72 | 62 | 98 | 44 | 69 | 14 | 46 | 61 |
| 18 | 11 | 8 | 7 | 7 | 10 | 101 | 82 | 52 | 60 | 102 | 32 | 40 | 42 | 56 | 59 |
| 19 | 5 | 7 | 6 | 5 | 8 | 88 | 96 | 58 | 60 | 100 | 46 | 61 | 59 | 59 | 61 |
| 20 | 12 | 14 | 6 | 5 | 7 | 64 | 76 | 70 | 76 | 72 | 55 | 60 | 71 | 55 | 58 |
| 21 | 10 | 7 | 7 | 9 | 10 | 100 | 92 | 58 | 76 | 92 |  |  |  |  | 53 |
| 22 | 6 | 7 | 8 | 8 | 8 | 102 | 62 | 70 | 54 | 94 |  |  |  |  |  |
| 23 | 6 | 9 | 7 | 8 | 9 | 100 | 92 | 60 | 62 | 80 |  |  |  |  |  |
| 24 | 12 | 10 | 6 | 12 | 7 | 86 | 80 | 74 | 66 | 98 |  |  |  |  |  |
| 25 | 9 | 12 | 7 | 10 | 10 | 89 | 60 | 68 | 60 | 88 |  |  |  |  |  |
| 26 | 8 | 7 | 7 | 10 | 9 | 123 | 88 | 72 | 58 | 122 |  |  |  |  |  |
| 27 | 8 | 10 | 5 | 7 | 7 | 104 | 92 | 56 | 68 | 108 |  |  |  |  |  |
| 28 | 10 | 9 | 6 | 9 | 6 | 102 | 100 | 62 | 58 | 92 |  |  |  |  |  |
| 29 | 11 | 10 | 6 | 8 | 10 | 96 | 103 | 68 | 74 | 76 |  |  |  |  |  |
| 30 | 11 | 11 | 10 | 10 | 8 | 90 | 96 | 62 | 68 |  |  |  |  |  |  |

Note: ctrl means *control group*; the unit of dose is *μL/mL*
