# Supplementary material for: Effects of commercial beverages on the neurobehavioral motility of Caenorhabditis elegans
Source: PeerJ. 2022 Jul 14;10:e13563. doi: 10.7717/peerj.13563 (PMC9288823; doi:10.7717/peerj.13563)
Supplement: Supplemental Information 8 [file peerj-10-13563-s008.docx]

**Table S8--raw data--Neurobehavioral changes of nematodes treated by brown carbonated drink**

| **No.** | **body bend** | | | | | **head thrash** | | | | | **pharyngeal pump** | | | | |
| --- | --- | --- | --- | --- | --- | --- | --- | --- | --- | --- | --- | --- | --- | --- | --- |
|  | 500 | 250 | 125 | 62.5 | ctr | 500 | 250 | 125 | 62.5 | ctr | 500 | 250 | 125 | 62.5 | ctr |
| 1 | 6 | 5 | 5 | 5 | 5 | 54 | 52 | 52 | 58 | 90 | 66 | 56 | 51 | 73 | 62 |
| 2 | 7 | 3 | 3 | 6 | 7 | 78 | 56 | 46 | 54 | 84 | 70 | 68 | 52 | 62 | 55 |
| 3 | 5 | 5 | 4 | 5 | 8 | 68 | 50 | 58 | 54 | 80 | 67 | 60 | 62 | 68 | 78 |
| 4 | 5 | 7 | 6 | 4 | 7 | 44 | 58 | 74 | 66 | 96 | 69 | 50 | 68 | 34 | 69 |
| 5 | 5 | 6 | 4 | 3 | 7 | 66 | 52 | 64 | 56 | 84 | 67 | 68 | 35 | 45 | 72 |
| 6 | 5 | 5 | 5 | 5 | 5 | 70 | 62 | 68 | 52 | 96 | 57 | 68 | 47 | 75 | 68 |
| 7 | 4 | 5 | 5 | 7 | 6 | 68 | 64 | 60 | 64 | 98 | 53 | 52 | 65 | 55 | 69 |
| 8 | 5 | 4 | 4 | 6 | 6 | 44 | 66 | 88 | 70 | 92 | 63 | 56 | 62 | 75 | 63 |
| 9 | 5 | 4 | 3 | 5 | 8 | 52 | 56 | 60 | 66 | 92 | 63 | 68 | 66 | 74 | 63 |
| 10 | 6 | 5 | 4 | 4 | 7 | 66 | 40 | 74 | 72 | 94 | 56 | 62 | 66 | 61 | 56 |
| 11 | 6 | 5 | 5 | 5 | 8 | 82 | 48 | 62 | 64 | 90 | 57 | 64 | 66 | 53 | 61 |
| 12 | 8 | 8 | 4 | 4 | 7 | 68 | 60 | 52 | 74 | 80 | 60 | 60 | 60 | 55 | 56 |
| 13 | 4 | 4 | 3 | 5 | 9 | 66 | 52 | 54 | 62 | 98 | 53 | 58 | 23 | 25 | 55 |
| 14 | 5 | 4 | 5 | 7 | 7 | 68 | 52 | 48 | 58 | 90 | 70 | 63 | 30 | 54 | 66 |
| 15 | 4 | 5 | 5 | 5 | 8 | 56 | 48 | 56 | 54 | 82 | 71 | 65 | 54 | 62 | 71 |
| 16 | 5 | 6 | 10 | 8 | 8 | 58 | 62 | 58 | 56 | 88 | 18 | 52 | 63 | 54 | 61 |
| 17 | 6 | 4 | 4 | 5 | 7 | 68 | 58 | 64 | 56 | 90 | 37 | 41 | 63 | 47 | 65 |
| 18 | 6 | 6 | 4 | 5 | 6 | 52 | 48 | 74 | 56 | 86 | 54 | 55 | 64 | 44 | 73 |
| 19 | 4 | 5 | 7 | 6 | 8 | 58 | 54 | 68 | 60 | 94 | 59 | 60 | 69 | 57 | 74 |
| 20 | 7 | 6 | 6 | 3 | 9 | 60 | 60 | 70 | 68 | 86 | 36 | 62 | 35 | 20 | 73 |
| 21 | 7 | 6 | 8 | 3 | 3 | 72 | 66 | 52 | 46 | 67 |  |  |  |  |  |
| 22 | 5 | 4 | 6 | 2 | 5 | 76 | 60 | 62 | 42 | 59 |  |  |  |  |  |
| 23 | 3 | 5 | 5 | 4 | 6 | 70 | 58 | 54 | 47 | 63 |  |  |  |  |  |
| 24 | 3 | 4 | 5 | 5 | 7 | 60 | 52 | 64 | 51 | 52 |  |  |  |  |  |
| 25 | 6 | 5 | 4 | 3 | 4 | 52 | 62 | 60 | 45 | 57 |  |  |  |  |  |
| 26 | 5 | 7 | 7 | 4 | 3 | 46 | 46 | 58 | 37 | 62 |  |  |  |  |  |
| 27 | 5 | 5 | 5 | 3 | 2 | 46 | 56 | 52 | 32 | 55 |  |  |  |  |  |
| 28 | 4 | 6 | 5 | 4 | 4 | 52 | 62 | 58 | 47 | 59 |  |  |  |  |  |
| 29 | 4 | 4 | 4 | 3 | 3 | 48 | 48 | 64 | 37 | 63 |  |  |  |  |  |
| 30 | 5 | 8 | 7 | 4 | 5 | 56 | 52 | 54 | 45 | 61 |  |  |  |  |  |

Note: ctrl means *control group*; the unit of dose is *μL/mL*
