# Supplementary material for: Effects of commercial beverages on the neurobehavioral motility of Caenorhabditis elegans
Source: PeerJ. 2022 Jul 14;10:e13563. doi: 10.7717/peerj.13563 (PMC9288823; doi:10.7717/peerj.13563)
Supplement: Supplemental Information 9 — raw data [file peerj-10-13563-s009.docx]

**Table S9--raw data--Neurobehavioral changes of nematodes treated by colorless carbonated drink**

| **No.** | **body bend** | | | | | **head thrash** | | | | | **pharyngeal pump** | | | | |
| --- | --- | --- | --- | --- | --- | --- | --- | --- | --- | --- | --- | --- | --- | --- | --- |
|  | 500 | 250 | 125 | 62.5 | ctr | 500 | 250 | 125 | 62.5 | ctr | 500 | 250 | 125 | 62.5 | ctr |
| 1 | 7 | 7 | 7 | 8 | 5 | 70 | 54 | 56 | 46 | 90 | 65 | 51 | 49 | 58 | 62 |
| 2 | 5 | 6 | 5 | 5 | 7 | 82 | 72 | 59 | 52 | 84 | 61 | 57 | 59 | 42 | 55 |
| 3 | 2 | 3 | 3 | 4 | 8 | 96 | 50 | 60 | 51 | 80 | 60 | 58 | 59 | 65 | 78 |
| 4 | 3 | 6 | 4 | 2 | 7 | 44 | 84 | 57 | 45 | 96 | 65 | 59 | 58 | 69 | 69 |
| 5 | 4 | 8 | 6 | 3 | 7 | 102 | 88 | 56 | 47 | 84 | 64 | 56 | 62 | 61 | 72 |
| 6 | 3 | 3 | 7 | 6 | 5 | 84 | 68 | 47 | 43 | 96 | 63 | 62 | 32 | 53 | 68 |
| 7 | 8 | 4 | 5 | 5 | 6 | 90 | 68 | 60 | 49 | 98 | 71 | 49 | 52 | 53 | 69 |
| 8 | 5 | 3 | 3 | 3 | 6 | 67 | 72 | 50 | 52 | 92 | 54 | 63 | 41 | 65 | 63 |
| 9 | 2 | 3 | 2 | 6 | 8 | 60 | 96 | 58 | 51 | 92 | 66 | 56 | 66 | 66 | 63 |
| 10 | 6 | 8 | 4 | 4 | 7 | 98 | 92 | 55 | 47 | 94 | 31 | 51 | 63 | 73 | 56 |
| 11 | 6 | 5 | 3 | 3 | 8 | 66 | 106 | 63 | 70 | 90 | 70 | 44 | 42 | 73 | 61 |
| 12 | 8 | 8 | 7 | 3 | 7 | 18 | 68 | 59 | 72 | 80 | 5 | 46 | 65 | 63 | 56 |
| 13 | 4 | 4 | 6 | 4 | 9 | 52 | 78 | 60 | 52 | 98 | 52 | 60 | 66 | 72 | 55 |
| 14 | 3 | 4 | 5 | 4 | 7 | 69 | 103 | 57 | 54 | 90 | 56 | 65 | 65 | 65 | 66 |
| 15 | 4 | 5 | 6 | 3 | 8 | 86 | 110 | 66 | 49 | 82 | 49 | 59 | 70 | 52 | 71 |
| 16 | 3 | 6 | 7 | 2 | 8 | 38 | 74 | 61 | 51 | 88 | 64 | 60 | 73 | 68 | 61 |
| 17 | 7 | 7 | 4 | 3 | 7 | 58 | 84 | 64 | 60 | 90 | 60 | 59 | 56 | 61 | 65 |
| 18 | 3 | 8 | 3 | 4 | 6 | 88 | 92 | 58 | 53 | 86 | 71 | 60 | 45 | 67 | 73 |
| 19 | 3 | 9 | 5 | 3 | 8 | 60 | 100 | 62 | 57 | 94 | 52 | 59 | 57 | 68 | 74 |
| 20 | 7 | 6 | 2 | 5 | 9 | 79 | 80 | 59 | 59 | 86 | 35 | 61 | 69 | 55 | 73 |
| 21 | 6 | 4 | 5 | 3 | 3 | 80 | 102 | 65 | 53 | 67 |  |  | 70 |  |  |
| 22 | 2 | 6 | 7 | 2 | 5 | 72 | 96 | 67 | 59 | 59 |  |  |  |  |  |
| 23 | 3 | 6 | 4 | 6 | 6 | 64 | 98 | 59 | 52 | 63 |  |  |  |  |  |
| 24 | 5 | 6 | 5 | 4 | 7 | 60 | 99 | 67 | 52 | 52 |  |  |  |  |  |
| 25 | 9 | 4 | 6 | 4 | 4 | 88 | 80 | 59 | 53 | 57 |  |  |  |  |  |
| 26 | 5 | 7 | 2 | 3 | 3 | 84 | 52 | 57 | 53 | 62 |  |  |  |  |  |
| 27 | 5 | 5 | 3 | 3 | 2 | 51 | 89 | 56 | 51 | 55 |  |  |  |  |  |
| 28 | 4 | 6 | 7 | 4 | 4 | 52 | 96 | 63 | 57 | 59 |  |  |  |  |  |
| 29 | 7 | 2 | 5 | 3 | 3 |  | 78 | 61 | 56 | 63 |  |  |  |  |  |
| 30 | 6 | 5 | 3 | 2 | 5 |  | 68 | 59 | 62 | 61 |  |  |  |  |  |

Note: ctrl means *control group*; the unit of dose is *μL/mL*
