# Supplementary material for: Effects of commercial beverages on the neurobehavioral motility of Caenorhabditis elegans
Source: PeerJ. 2022 Jul 14;10:e13563. doi: 10.7717/peerj.13563 (PMC9288823; doi:10.7717/peerj.13563)
Supplement: Supplemental Information 10 [file peerj-10-13563-s010.docx]

**Table S10--raw data--Neurobehavioral changes of nematodes treated by orange carbonated drink**

| **No.** | **body bend** | | | | | **head thrash** | | | | | **pharyngeal pump** | | | | |
| --- | --- | --- | --- | --- | --- | --- | --- | --- | --- | --- | --- | --- | --- | --- | --- |
|  | 500 | 250 | 125 | 62.5 | ctr | 500 | 250 | 125 | 62.5 | ctr | 500 | 250 | 125 | 62.5 | ctr |
| 1 | 8 | 7 | 5 | 8 | 5 | 50 | 88 | 56 | 84 | 90 | 43 | 51 | 60 | 69 | 62 |
| 2 | 4 | 8 | 6 | 10 | 7 | 44 | 94 | 30 | 92 | 84 | 39 | 58 | 63 | 70 | 55 |
| 3 | 7 | 7 | 10 | 9 | 8 | 40 | 92 | 75 | 98 | 80 | 61 | 63 | 64 | 46 | 78 |
| 4 | 3 | 8 | 11 | 7 | 7 | 47 | 86 | 64 | 80 | 96 | 56 | 55 | 5 | 39 | 69 |
| 5 | 4 | 6 | 5 | 6 | 7 | 46 | 72 | 82 | 94 | 84 | 50 | 67 | 45 | 64 | 72 |
| 6 | 5 | 9 | 7 | 8 | 5 | 41 | 96 | 32 | 84 | 96 | 58 | 65 | 48 | 43 | 68 |
| 7 | 4 | 9 | 7 | 8 | 6 | 56 | 80 | 24 | 80 | 98 | 60 | 70 | 52 | 67 | 69 |
| 8 | 5 | 8 | 7 | 7 | 6 | 44 | 90 | 21 | 92 | 92 | 66 | 53 | 71 | 66 | 63 |
| 9 | 7 | 10 | 6 | 7 | 8 | 52 | 92 | 80 | 88 | 92 | 70 | 69 | 48 | 66 | 63 |
| 10 | 2 | 8 | 4 | 10 | 7 | 50 | 94 | 52 | 82 | 94 | 64 | 62 | 70 | 65 | 56 |
| 11 | 10 | 9 | 6 | 8 | 8 | 60 | 64 | 62 | 86 | 90 | 46 | 45 | 60 | 58 | 61 |
| 12 | 5 | 8 | 4 | 10 | 7 | 55 | 88 | 60 | 94 | 80 | 53 | 60 | 53 | 43 | 56 |
| 13 | 6 | 6 | 7 | 12 | 9 | 51 | 106 | 44 | 92 | 98 | 60 | 71 | 64 | 73 | 55 |
| 14 | 3 | 7 | 3 | 11 | 7 | 53 | 104 | 39 | 82 | 90 | 64 | 47 | 68 | 53 | 66 |
| 15 | 4 | 8 | 6 | 8 | 8 | 49 | 98 | 60 | 84 | 82 | 59 | 10 | 69 | 24 | 71 |
| 16 | 3 | 10 | 5 | 9 | 8 | 49 | 90 | 44 | 82 | 88 | 42 | 50 | 76 | 57 | 61 |
| 17 | 4 | 7 | 7 | 8 | 7 | 48 | 88 | 48 | 90 | 90 | 56 | 62 | 58 | 56 | 65 |
| 18 | 4 | 10 | 6 | 10 | 6 | 47 | 92 | 65 | 100 | 86 | 50 | 62 | 55 | 62 | 73 |
| 19 | 4 | 8 | 5 | 8 | 8 | 46 | 90 | 29 | 92 | 94 | 62 | 65 | 70 | 61 | 74 |
| 20 | 2 | 7 | 5 | 6 | 9 | 43 | 92 | 68 | 98 | 86 | 63 | 45 | 41 | 42 | 73 |
| 21 | 8 | 10 | 7 | 7 | 3 | 53 | 100 | 92 | 98 | 67 |  |  |  |  |  |
| 22 | 7 | 8 | 8 | 5 | 5 | 56 | 84 | 120 | 94 | 59 |  |  |  |  |  |
| 23 | 5 | 7 | 6 | 7 | 6 | 54 | 76 | 55 | 86 | 63 |  |  |  |  |  |
| 24 | 5 | 10 | 7 | 8 | 7 | 51 | 100 | 70 | 76 | 52 |  |  |  |  |  |
| 25 | 4 | 9 | 4 | 8 | 4 | 42 | 86 | 54 | 86 | 57 |  |  |  |  |  |
| 26 | 3 | 7 | 4 | 10 | 3 | 48 | 82 | 80 | 74 | 62 |  |  |  |  |  |
| 27 | 3 | 8 | 5 | 9 | 2 | 53 | 86 | 78 | 86 | 55 |  |  |  |  |  |
| 28 | 4 | 8 | 10 | 5 | 4 | 55 | 96 | 68 | 78 | 59 |  |  |  |  |  |
| 29 | 5 | 9 | 5 | 9 | 3 | 52 | 84 | 78 | 76 | 63 |  |  |  |  |  |
| 30 | 3 | 8 | 7 | 6 | 5 | 53 | 90 | 62 | 80 | 61 |  |  |  |  |  |

Note: ctrl means *control group*; the unit of dose is *μL/mL*
