# Supplementary material for: Effects of commercial beverages on the neurobehavioral motility of Caenorhabditis elegans
Source: PeerJ. 2022 Jul 14;10:e13563. doi: 10.7717/peerj.13563 (PMC9288823; doi:10.7717/peerj.13563)
Supplement: Supplemental Information 11 [file peerj-10-13563-s011.docx]

**Table S11--raw data--Neurobehavioral changes of nematodes treated by sports functional drink**

| **No.** | **body bend** | | | | | **head thrash** | | | | | **pharyngeal pump** | | | | |
| --- | --- | --- | --- | --- | --- | --- | --- | --- | --- | --- | --- | --- | --- | --- | --- |
|  | 500 | 250 | 125 | 62.5 | ctr | 500 | 250 | 125 | 62.5 | ctr | 500 | 250 | 125 | 62.5 | ctr |
| 1 | 7 | 7 | 8 | 4 | 3 | 118 | 89 | 52 | 54 | 62 | 53 | 40 | 42 | 57 | 55 |
| 2 | 5 | 4 | 10 | 5 | 4 | 104 | 97 | 52 | 60 | 64 | 59 | 51 | 70 | 58 | 58 |
| 3 | 4 | 5 | 10 | 6 | 4 | 100 | 100 | 55 | 64 | 60 | 59 | 53 | 61 | 47 | 57 |
| 4 | 3 | 6 | 7 | 5 | 4 | 98 | 90 | 50 | 66 | 54 | 56 | 50 | 54 | 66 | 56 |
| 5 | 4 | 5 | 9 | 8 | 6 | 112 | 96 | 49 | 66 | 52 | 53 | 36 | 54 | 61 | 61 |
| 6 | 4 | 12 | 12 | 5 | 4 | 45 | 64 | 48 | 68 | 52 | 58 | 63 | 45 | 52 | 62 |
| 7 | 5 | 5 | 9 | 4 | 6 | 80 | 98 | 49 | 62 | 50 | 49 | 59 | 51 | 17 | 43 |
| 8 | 9 | 4 | 8 | 3 | 3 | 100 | 66 | 51 | 56 | 50 | 23 | 34 | 42 | 55 | 59 |
| 9 | 3 | 3 | 11 | 6 | 4 | 50 | 58 | 49 | 44 | 48 | 49 | 46 | 55 | 54 | 57 |
| 10 | 5 | 6 | 10 | 5 | 3 | 72 | 97 | 53 | 52 | 62 | 56 | 71 | 55 | 58 | 42 |
| 11 | 8 | 4 | 4 | 4 | 4 | 92 | 82 | 66 | 60 | 60 | 61 | 47 | 43 | 37 | 54 |
| 12 | 6 | 6 | 4 | 3 | 6 | 111 | 86 | 58 | 52 | 60 | 61 | 61 | 2 | 43 | 50 |
| 13 | 5 | 9 | 3 | 3 | 5 | 110 | 78 | 70 | 48 | 64 | 62 | 55 | 43 | 39 | 49 |
| 14 | 9 | 7 | 5 | 8 | 3 | 96 | 74 | 44 | 58 | 70 | 61 | 45 | 49 | 51 | 56 |
| 15 | 5 | 8 | 4 | 6 | 4 | 96 | 106 | 60 | 56 | 66 | 54 | 45 | 43 | 48 | 54 |
| 16 | 7 | 6 | 5 | 6 | 6 | 102 | 56 | 42 | 54 | 70 | 50 | 52 | 49 | 48 | 50 |
| 17 | 5 | 9 | 6 | 7 | 3 | 98 | 64 | 54 | 62 | 58 | 63 | 56 | 67 | 51 | 52 |
| 18 | 6 | 5 | 5 | 5 | 4 | 99 | 78 | 56 | 70 | 62 | 48 | 49 | 43 | 55 | 48 |
| 19 | 7 | 10 | 9 | 5 | 4 | 72 | 82 | 52 | 50 | 60 | 59 | 53 | 0 | 53 | 26 |
| 20 | 5 | 7 | 7 | 4 | 5 | 103 | 86 | 60 | 66 | 60 | 70 | 63 | 61 | 54 | 40 |
| 21 | 8 | 6 | 7 | 3 | 6 | 86 | 96 | 94 | 68 | 44 | 61 |  |  |  |  |
| 22 | 4 | 5 | 3 | 3 | 3 | 110 | 58 | 100 | 52 | 60 |  |  |  |  |  |
| 23 | 6 | 5 | 5 | 6 | 4 | 99 | 114 | 104 | 70 | 56 |  |  |  |  |  |
| 24 | 7 | 9 | 3 | 5 | 3 | 82 | 74 | 98 | 62 | 70 |  |  |  |  |  |
| 25 | 8 | 8 | 6 | 7 | 8 | 68 | 104 | 94 | 74 | 60 |  |  |  |  |  |
| 26 | 6 | 8 | 7 | 6 | 7 | 94 | 64 | 96 | 54 | 64 |  |  |  |  |  |
| 27 | 6 | 7 | 8 | 7 | 5 | 46 | 47 | 102 | 66 | 56 |  |  |  |  |  |
| 28 | 9 | 7 | 3 | 5 | 4 | 70 | 76 | 98 | 58 | 76 |  |  |  |  |  |
| 29 | 8 | 6 | 3 | 4 | 5 | 97 | 78 | 88 | 72 | 68 |  |  |  |  |  |
| 30 | 11 | 4 | 4 | 5 | 6 | 78 | 102 | 92 | 50 | 64 |  |  |  |  |  |

Note: ctrl means *control group*; the unit of dose is *μL/mL*
