# Supplementary material for: Effects of commercial beverages on the neurobehavioral motility of Caenorhabditis elegans
Source: PeerJ. 2022 Jul 14;10:e13563. doi: 10.7717/peerj.13563 (PMC9288823; doi:10.7717/peerj.13563)
Supplement: Supplemental Information 12 [file peerj-10-13563-s012.docx]

**Table S12--raw data--Neurobehavioral changes of nematodes treated by fatigue relieving functional drink**

| **No.** | **body bend** | | | | | **head thrash** | | | | | **pharyngeal pump** | | | | |
| --- | --- | --- | --- | --- | --- | --- | --- | --- | --- | --- | --- | --- | --- | --- | --- |
|  | 500 | 250 | 125 | 62.5 | ctr | 500 | 250 | 125 | 62.5 | ctr | 500 | 250 | 125 | 62.5 | ctr |
| 1 | 1 | 5 | 5 | 5 | 8 | 23 | 38 | 78 | 64 | 50 | 35 | 49 | 39 | 33 | 62 |
| 2 | 2 | 3 | 6 | 4 | 5 | 24 | 37 | 84 | 45 | 46 | 39 | 34 | 51 | 36 | 0 |
| 3 | 1 | 2 | 4 | 5 | 8 | 24 | 36 | 46 | 52 | 90 | 32 | 40 | 36 | 30 | 37 |
| 4 | 2 | 2 | 7 | 5 | 8 | 34 | 41 | 52 | 68 | 91 | 30 | 37 | 42 | 36 | 77 |
| 5 | 3 | 2 | 6 | 6 | 9 | 28 | 39 | 51 | 86 | 66 | 45 | 46 | 44 | 58 | 69 |
| 6 | 2 | 3 | 8 | 4 | 8 | 23 | 40 | 84 | 58 | 88 | 33 | 37 | 35 | 41 | 53 |
| 7 | 1 | 4 | 10 | 7 | 9 | 25 | 37 | 56 | 54 | 74 | 30 | 44 | 34 | 43 | 36 |
| 8 | 1 | 3 | 6 | 3 | 7 | 30 | 43 | 72 | 70 | 88 | 41 | 37 | 50 | 34 | 65 |
| 9 | 2 | 2 | 3 | 4 | 6 | 32 | 45 | 90 | 48 | 84 | 35 | 42 | 21 | 47 | 55 |
| 10 | 1 | 1 | 6 | 4 | 8 | 23 | 49 | 68 | 90 | 44 | 39 | 42 | 45 | 42 | 76 |
| 11 | 1 | 5 | 5 | 8 | 6 | 45 | 63 | 74 | 70 | 74 | 33 | 52 | 51 | 42 | 74 |
| 12 | 1 | 8 | 4 | 5 | 7 | 46 | 62 | 80 | 74 | 80 | 35 | 40 | 45 | 36 | 42 |
| 13 | 3 | 4 | 4 | 8 | 5 | 55 | 55 | 45 | 80 | 78 | 40 | 34 | 36 | 45 | 63 |
| 14 | 2 | 7 | 5 | 4 | 9 | 50 | 54 | 66 | 88 | 80 | 35 | 40 | 40 | 36 | 32 |
| 15 | 1 | 4 | 8 | 6 | 6 | 48 | 42 | 56 | 56 | 76 | 41 | 53 | 44 | 34 | 77 |
| 16 | 1 | 2 | 5 | 4 | 7 | 48 | 47 | 72 | 90 | 54 | 43 | 52 | 43 | 37 | 45 |
| 17 | 5 | 3 | 5 | 3 | 7 | 40 | 53 | 56 | 56 | 72 | 45 | 41 | 40 | 40 | 71 |
| 18 | 2 | 4 | 5 | 5 | 6 | 31 | 40 | 94 | 88 | 84 | 35 | 44 | 52 | 39 | 40 |
| 19 | 1 | 5 | 6 | 5 | 5 | 43 | 52 | 98 | 80 | 58 | 44 | 42 | 52 | 49 | 63 |
| 20 | 2 | 3 | 3 | 5 | 5 | 54 | 44 | 68 | 43 | 54 | 45 | 45 | 41 | 43 | 35 |
| 21 | 4 | 6 | 6 | 8 | 8 | 28 | 56 | 76 | 66 | 60 | 38 |  |  |  |  |
| 22 | 4 | 5 | 7 | 6 | 6 | 59 | 59 | 90 | 70 | 86 | 33 |  |  |  |  |
| 23 | 2 | 8 | 5 | 7 | 5 | 52 | 55 | 102 | 62 | 78 | 43 |  |  |  |  |
| 24 | 2 | 4 | 4 | 5 | 7 | 58 | 61 | 92 | 72 | 45 | 36 |  |  |  |  |
| 25 | 3 | 6 | 6 | 3 | 5 | 60 | 54 | 76 | 42 | 84 | 47 |  |  |  |  |
| 26 | 2 | 4 | 5 | 4 | 5 | 40 | 55 | 92 | 54 | 72 | 50 |  |  |  |  |
| 27 | 3 | 5 | 7 | 4 | 6 | 50 | 57 | 84 | 78 | 68 |  |  |  |  |  |
| 28 | 2 | 3 | 6 | 10 | 4 | 49 | 54 | 78 | 84 | 74 |  |  |  |  |  |
| 29 | 2 | 7 | 8 | 5 | 9 | 55 | 52 | 66 | 64 | 88 |  |  |  |  |  |
| 30 | 2 | 4 | 7 | 9 | 7 | 21 | 56 | 58 | 42 | 64 |  |  |  |  |  |

Note: ctrl means *control group*; the unit of dose is *μL/mL*
