# Supplementary material for: Effects of commercial beverages on the neurobehavioral motility of Caenorhabditis elegans
Source: PeerJ. 2022 Jul 14;10:e13563. doi: 10.7717/peerj.13563 (PMC9288823; doi:10.7717/peerj.13563)
Supplement: Supplemental Information 13 [file peerj-10-13563-s013.docx]

**Table S13--raw data--Neurobehavioral changes of nematodes treated by black tea beverage**

| **No.** | **body bend** | | | | | **head thrash** | | | | | **pharyngeal pump** | | | | |
| --- | --- | --- | --- | --- | --- | --- | --- | --- | --- | --- | --- | --- | --- | --- | --- |
|  | 500 | 250 | 125 | 62.5 | ctr | 500 | 250 | 125 | 62.5 | ctr | 500 | 250 | 125 | 62.5 | ctr |
| 1 | 6 | 12 | 3 | 3 | 3 | 86 | 80 | 31 | 50 | 62 | 5 | 52 | 49 | 53 | 55 |
| 2 | 6 | 8 | 5 | 2 | 4 | 88 | 88 | 57 | 41 | 64 | 25 | 48 | 55 | 53 | 58 |
| 3 | 4 | 7 | 3 | 4 | 4 | 94 | 98 | 15 | 58 | 60 | 47 | 48 | 48 | 52 | 57 |
| 4 | 7 | 6 | 4 | 2 | 4 | 84 | 86 | 36 | 26 | 54 | 46 | 59 | 51 | 51 | 56 |
| 5 | 8 | 8 | 4 | 3 | 6 | 86 | 104 | 47 | 50 | 52 | 44 | 51 | 50 | 46 | 61 |
| 6 | 6 | 7 | 5 | 4 | 4 | 90 | 100 | 60 | 50 | 52 | 40 | 43 | 43 | 50 | 62 |
| 7 | 10 | 8 | 3 | 3 | 6 | 84 | 84 | 39 | 41 | 50 | 45 | 46 | 49 | 55 | 43 |
| 8 | 6 | 7 | 4 | 4 | 3 | 98 | 90 | 45 | 39 | 50 | 40 | 44 | 42 | 52 | 59 |
| 9 | 10 | 9 | 5 | 5 | 4 | 90 | 98 | 24 | 47 | 48 | 9 | 52 | 46 | 45 | 57 |
| 10 | 6 | 7 | 3 | 4 | 3 | 78 | 100 | 37 | 46 | 62 | 33 | 40 | 36 | 51 | 42 |
| 11 | 10 | 11 | 6 | 2 | 4 | 104 | 92 | 55 | 39 | 60 | 34 | 53 | 44 | 41 | 54 |
| 12 | 7 | 10 | 2 | 5 | 6 | 102 | 84 | 57 | 50 | 60 | 37 | 43 | 54 | 38 | 50 |
| 13 | 5 | 12 | 5 | 3 | 5 | 104 | 94 | 47 | 49 | 64 | 28 | 33 | 47 | 45 | 49 |
| 14 | 8 | 9 | 4 | 6 | 3 | 86 | 86 | 51 | 44 | 70 | 43 | 48 | 48 | 46 | 56 |
| 15 | 7 | 8 | 4 | 3 | 4 | 90 | 80 | 39 | 48 | 66 | 44 | 38 | 50 | 47 | 54 |
| 16 | 7 | 5 | 5 | 5 | 6 | 88 | 78 | 43 | 43 | 70 | 13 | 52 | 58 | 57 | 50 |
| 17 | 5 | 7 | 5 | 3 | 3 | 100 | 80 | 47 | 47 | 58 | 40 | 50 | 55 | 46 | 52 |
| 18 | 10 | 10 | 3 | 4 | 4 | 94 | 84 | 42 | 49 | 62 | 38 | 42 | 46 | 47 | 48 |
| 19 | 10 | 6 | 4 | 3 | 4 | 88 | 96 | 57 | 45 | 60 | 12 | 51 | 37 | 58 | 26 |
| 20 | 10 | 12 | 3 | 6 | 5 | 86 | 102 | 47 | 47 | 60 | 36 | 45 | 44 | 54 | 40 |
| 21 | 7 | 9 | 3 | 3 | 6 | 106 | 84 | 32 | 52 | 44 | 39 | 37 | 42 | 23 |  |
| 22 | 4 | 8 | 3 | 4 | 3 | 102 | 100 | 37 | 45 | 60 |  |  | 41 |  |  |
| 23 | 7 | 13 | 4 | 3 | 4 | 90 | 90 | 48 | 51 | 56 |  |  | 49 |  |  |
| 24 | 6 | 9 | 2 | 2 | 3 | 88 | 94 | 42 | 52 | 70 |  |  | 49 |  |  |
| 25 | 7 | 6 | 3 | 8 | 8 | 82 | 102 | 27 | 49 | 60 |  |  |  |  |  |
| 26 | 10 | 8 | 4 | 5 | 7 | 108 | 80 | 39 | 35 | 64 |  |  |  |  |  |
| 27 | 9 | 5 | 7 | 5 | 5 | 98 | 86 | 34 | 48 | 56 |  |  |  |  |  |
| 28 | 7 | 9 | 5 | 6 | 4 | 92 | 78 | 47 | 52 | 76 |  |  |  |  |  |
| 29 | 5 | 10 | 3 | 3 | 5 | 80 | 84 | 52 | 47 | 68 |  |  |  |  |  |
| 30 | 6 | 8 | 3 | 9 | 6 | 84 | 88 | 41 | 38 | 64 |  |  |  |  |  |

Note: ctrl means *control group*; the unit of dose is *μL/mL*
