# Supplementary material for: Effects of commercial beverages on the neurobehavioral motility of Caenorhabditis elegans
Source: PeerJ. 2022 Jul 14;10:e13563. doi: 10.7717/peerj.13563 (PMC9288823; doi:10.7717/peerj.13563)
Supplement: Supplemental Information 14 [file peerj-10-13563-s014.docx]

**Table S14--raw data--Neurobehavioral changes of nematodes treated by green tea beverage**

| **No.** | **body bend** | | | | | **head thrash** | | | | | **pharyngeal pump** | | | | |
| --- | --- | --- | --- | --- | --- | --- | --- | --- | --- | --- | --- | --- | --- | --- | --- |
|  | 500 | 250 | 125 | 62.5 | ctr | 500 | 250 | 125 | 62.5 | ctr | 500 | 250 | 125 | 62.5 | ctr |
| 1 | 8 | 5 | 5 | 5 | 3 | 64 | 54 | 66 | 60 | 45 | 20 | 61 | 72 | 50 | 55 |
| 2 | 5 | 4 | 5 | 7 | 4 | 49 | 44 | 62 | 66 | 42 | 34 | 56 | 57 | 48 | 60 |
| 3 | 3 | 4 | 5 | 4 | 6 | 58 | 62 | 64 | 58 | 50 | 53 | 44 | 50 | 53 | 59 |
| 4 | 3 | 5 | 6 | 6 | 7 | 45 | 53 | 58 | 52 | 48 | 43 | 46 | 58 | 45 | 54 |
| 5 | 5 | 4 | 3 | 5 | 7 | 42 | 37 | 74 | 62 | 52 | 32 | 52 | 40 | 46 | 63 |
| 6 | 4 | 5 | 4 | 4 | 6 | 44 | 56 | 48 | 62 | 49 | 41 | 60 | 55 | 65 | 66 |
| 7 | 3 | 3 | 3 | 6 | 8 | 50 | 48 | 70 | 64 | 53 | 32 | 53 | 40 | 67 | 58 |
| 8 | 5 | 5 | 9 | 8 | 9 | 65 | 61 | 64 | 70 | 48 | 49 | 51 | 42 | 38 | 60 |
| 9 | 4 | 4 | 8 | 5 | 7 | 36 | 45 | 54 | 66 | 62 | 46 | 64 | 41 | 45 | 55 |
| 10 | 5 | 4 | 4 | 6 | 6 | 50 | 44 | 66 | 68 | 59 | 40 | 53 | 30 | 43 | 69 |
| 11 | 6 | 5 | 5 | 8 | 9 | 57 | 63 | 56 | 64 | 46 | 60 | 57 | 45 | 35 | 68 |
| 12 | 4 | 4 | 4 | 4 | 6 | 64 | 59 | 66 | 48 | 49 | 56 | 59 | 44 | 36 | 54 |
| 13 | 9 | 4 | 3 | 6 | 7 | 56 | 50 | 64 | 68 | 45 | 58 | 50 | 52 | 37 | 42 |
| 14 | 7 | 4 | 2 | 8 | 8 | 56 | 52 | 62 | 60 | 53 | 45 | 59 | 51 | 45 | 46 |
| 15 | 6 | 8 | 3 | 4 | 6 | 48 | 48 | 60 | 58 | 48 | 54 | 57 | 50 | 59 | 52 |
| 16 | 11 | 6 | 6 | 7 | 7 | 47 | 49 | 66 | 58 | 49 | 51 | 51 | 64 | 54 | 44 |
| 17 | 6 | 6 | 3 | 7 | 5 | 53 | 60 | 68 | 54 | 52 | 52 | 50 | 44 | 44 | 56 |
| 18 | 5 | 3 | 2 | 7 | 6 | 51 | 57 | 54 | 62 | 51 | 59 | 48 | 44 | 45 | 50 |
| 19 | 3 | 6 | 6 | 5 | 8 | 42 | 51 | 48 | 60 | 47 | 52 | 47 | 59 | 59 | 45 |
| 20 | 4 | 5 | 4 | 3 | 7 | 45 | 50 | 58 | 54 | 46 | 49 | 60 | 54 | 54 | 56 |
| 21 | 4 | 9 | 6 | 4 | 6 | 59 | 58 | 74 | 54 | 47 |  |  |  |  |  |
| 22 | 4 | 7 | 5 | 5 | 6 | 54 | 56 | 66 | 60 | 46 |  |  |  |  |  |
| 23 | 3 | 5 | 6 | 3 | 7 | 68 | 59 | 58 | 64 | 51 |  |  |  |  |  |
| 24 | 5 | 8 | 3 | 5 | 5 | 56 | 64 | 52 | 48 | 45 |  |  |  |  |  |
| 25 | 5 | 11 | 3 | 7 | 8 | 72 | 44 | 54 | 60 | 56 |  |  |  |  |  |
| 26 | 4 | 4 | 7 | 9 | 7 | 56 | 61 | 58 | 74 | 50 |  |  |  |  |  |
| 27 | 4 | 7 | 4 | 6 | 8 | 49 | 52 | 60 | 60 | 47 |  |  |  |  |  |
| 28 | 6 | 8 | 3 | 5 | 5 | 55 | 58 | 60 | 46 | 44 |  |  |  |  |  |
| 29 | 6 | 7 | 4 | 3 | 6 | 50 | 55 | 48 | 58 | 43 |  |  |  |  |  |
| 30 | 5 | 6 | 8 | 9 | 6 | 54 | 53 | 58 | 59 | 49 |  |  |  |  |  |

Note: ctrl means *control group*; the unit of dose is *μL/mL*
