# Supplementary material for: Effects of commercial beverages on the neurobehavioral motility of Caenorhabditis elegans
Source: PeerJ. 2022 Jul 14;10:e13563. doi: 10.7717/peerj.13563 (PMC9288823; doi:10.7717/peerj.13563)
Supplement: Supplemental Information 15 [file peerj-10-13563-s015.docx]

**Table S15--raw data--Neurobehavioral changes of nematodes treated by coffee drink**

| **No.** | **body bend** | | | | | **head thrash** | | | | | **pharyngeal pump** | | | | |
| --- | --- | --- | --- | --- | --- | --- | --- | --- | --- | --- | --- | --- | --- | --- | --- |
|  | 500 | 250 | 125 | 62.5 | ctr | 500 | 250 | 125 | 62.5 | ctr | 500 | 250 | 125 | 62.5 | ctr |
| 1 | 4 | 4 | 7 | 6 | 3 | 2 | 53 | 57 | 53 | 45 | 60 | 49 | 55 | 53 | 55 |
| 2 | 5 | 6 | 8 | 4 | 4 | 42 | 50 | 51 | 56 | 42 | 56 | 51 | 43 | 59 | 60 |
| 3 | 4 | 6 | 4 | 6 | 6 | 58 | 51 | 51 | 45 | 50 | 60 | 51 | 47 | 50 | 59 |
| 4 | 11 | 5 | 7 | 5 | 7 | 56 | 52 | 50 | 51 | 48 | 44 | 59 | 38 | 41 | 54 |
| 5 | 5 | 7 | 5 | 2 | 7 | 50 | 57 | 49 | 48 | 52 | 53 | 34 | 41 | 57 | 63 |
| 6 | 4 | 7 | 5 | 7 | 6 | 49 | 52 | 53 | 51 | 49 | 57 | 49 | 58 | 58 | 66 |
| 7 | 5 | 6 | 7 | 8 | 8 | 52 | 52 | 55 | 49 | 53 | 52 | 49 | 48 | 42 | 58 |
| 8 | 3 | 5 | 7 | 5 | 9 | 48 | 53 | 55 | 59 | 48 | 70 | 62 | 53 | 50 | 60 |
| 9 | 2 | 4 | 3 | 7 | 7 | 34 | 47 | 56 | 47 | 62 | 47 | 53 | 38 | 69 | 55 |
| 10 | 4 | 6 | 4 | 4 | 6 | 37 | 50 | 54 | 43 | 59 | 58 | 42 | 45 | 50 | 69 |
| 11 | 4 | 8 | 8 | 5 | 9 | 55 | 53 | 48 | 49 | 46 | 68 | 48 | 30 | 60 | 68 |
| 12 | 9 | 7 | 4 | 8 | 6 | 52 | 47 | 50 | 40 | 49 | 61 | 52 | 39 | 47 | 54 |
| 13 | 6 | 9 | 7 | 6 | 7 | 47 | 48 | 49 | 40 | 45 | 47 | 56 | 47 | 60 | 42 |
| 14 | 6 | 7 | 5 | 6 | 8 | 56 | 45 | 46 | 38 | 53 | 60 | 48 | 42 | 54 | 46 |
| 15 | 8 | 6 | 5 | 5 | 6 | 54 | 50 | 46 | 38 | 48 | 59 | 50 | 39 | 36 | 52 |
| 16 | 7 | 9 | 3 | 4 | 7 | 49 | 47 | 47 | 42 | 49 | 59 | 40 | 36 | 35 | 44 |
| 17 | 4 | 5 | 6 | 5 | 5 | 55 | 52 | 42 | 44 | 52 | 62 | 40 | 60 | 60 | 56 |
| 18 | 5 | 5 | 5 | 4 | 6 | 57 | 42 | 47 | 38 | 51 | 51 | 33 | 40 | 43 | 50 |
| 19 | 7 | 6 | 4 | 4 | 8 | 51 | 44 | 44 | 36 | 47 | 50 | 45 | 56 | 40 | 45 |
| 20 | 6 | 5 | 7 | 5 | 7 | 54 | 53 | 49 | 38 | 46 | 49 | 45 | 52 | 45 | 56 |
| 21 | 7 | 3 | 6 | 5 | 6 | 30 | 64 | 44 | 50 | 47 |  |  |  |  |  |
| 22 | 5 | 4 | 8 | 6 | 6 | 44 | 50 | 46 | 47 | 46 |  |  |  |  |  |
| 23 | 4 | 7 | 5 | 6 | 7 | 37 | 54 | 42 | 52 | 51 |  |  |  |  |  |
| 24 | 4 | 4 | 7 | 4 | 5 | 38 | 46 | 47 | 50 | 45 |  |  |  |  |  |
| 25 | 5 | 4 | 4 | 8 | 8 | 49 | 49 | 52 | 51 | 56 |  |  |  |  |  |
| 26 | 2 | 5 | 6 | 6 | 7 | 40 | 41 | 35 | 54 | 50 |  |  |  |  |  |
| 27 | 4 | 7 | 6 | 3 | 8 | 36 | 50 | 28 | 56 | 47 |  |  |  |  |  |
| 28 | 6 | 4 | 5 | 4 | 5 | 40 | 40 | 39 | 45 | 44 |  |  |  |  |  |
| 29 | 3 | 5 | 6 | 3 | 6 | 46 | 54 | 34 | 42 | 43 |  |  |  |  |  |
| 30 | 4 | 5 | 4 | 4 | 6 | 48 | 46 | 42 | 52 | 49 |  |  |  |  |  |

Note: ctrl means *control group*; the unit of dose is *μL/mL*
