# Supplementary material for: Effects of commercial beverages on the neurobehavioral motility of Caenorhabditis elegans
Source: PeerJ. 2022 Jul 14;10:e13563. doi: 10.7717/peerj.13563 (PMC9288823; doi:10.7717/peerj.13563)
Supplement: Supplemental Information 16 [file peerj-10-13563-s016.docx]

**Table S16--raw data--Neurobehavioral changes of nematodes treated by herbal tea drink**

| **No.** | **body bend** | | | | | **head thrash** | | | | | **pharyngeal pump** | | | | |
| --- | --- | --- | --- | --- | --- | --- | --- | --- | --- | --- | --- | --- | --- | --- | --- |
|  | 500 | 250 | 125 | 62.5 | ctr | 500 | 250 | 125 | 62.5 | ctr | 500 | 250 | 125 | 62.5 | ctr |
| 1 | 9 | 8 | 9 | 6 | 3 | 38 | 90 | 41 | 81 | 45 | 42 | 44 | 52 | 63 | 55 |
| 2 | 6 | 8 | 7 | 8 | 4 | 34 | 74 | 45 | 51 | 42 | 40 | 59 | 37 | 65 | 60 |
| 3 | 6 | 6 | 8 | 7 | 6 | 34 | 56 | 47 | 68 | 50 | 36 | 61 | 50 | 75 | 59 |
| 4 | 4 | 9 | 6 | 7 | 7 | 33 | 76 | 49 | 88 | 48 | 54 | 44 | 56 | 36 | 54 |
| 5 | 8 | 5 | 5 | 7 | 7 | 36 | 94 | 48 | 84 | 52 | 40 | 50 | 60 | 62 | 63 |
| 6 | 6 | 6 | 10 | 7 | 6 | 43 | 95 | 50 | 81 | 49 | 40 | 31 | 51 | 62 | 66 |
| 7 | 5 | 8 | 9 | 7 | 8 | 45 | 79 | 47 | 81 | 53 | 49 | 48 | 42 | 53 | 58 |
| 8 | 7 | 8 | 7 | 9 | 9 | 43 | 91 | 46 | 76 | 48 | 50 | 50 | 46 | 74 | 60 |
| 9 | 9 | 8 | 8 | 5 | 7 | 47 | 83 | 43 | 52 | 62 | 47 | 37 | 46 | 51 | 55 |
| 10 | 5 | 7 | 10 | 7 | 6 | 48 | 67 | 42 | 53 | 59 | 59 | 44 | 61 | 57 | 69 |
| 11 | 6 | 9 | 4 | 8 | 9 | 41 | 65 | 47 | 85 | 46 | 58 | 51 | 52 | 59 | 68 |
| 12 | 6 | 7 | 9 | 8 | 6 | 35 | 99 | 40 | 83 | 49 | 53 | 47 | 37 | 61 | 54 |
| 13 | 7 | 9 | 5 | 10 | 7 | 40 | 79 | 46 | 75 | 45 | 43 | 39 | 59 | 50 | 42 |
| 14 | 6 | 8 | 6 | 8 | 8 | 40 | 41 | 43 | 80 | 53 | 39 | 47 | 57 | 49 | 46 |
| 15 | 10 | 8 | 4 | 11 | 6 | 37 | 62 | 39 | 81 | 48 | 37 | 62 | 40 | 47 | 52 |
| 16 | 6 | 7 | 4 | 5 | 7 | 39 | 55 | 50 | 59 | 49 | 52 | 52 | 35 | 40 | 44 |
| 17 | 7 | 10 | 5 | 6 | 5 | 46 | 51 | 41 | 63 | 52 | 42 | 37 | 47 | 37 | 56 |
| 18 | 10 | 6 | 9 | 5 | 6 | 46 | 61 | 43 | 66 | 51 | 54 | 60 | 59 | 43 | 50 |
| 19 | 9 | 8 | 10 | 7 | 8 | 42 | 47 | 40 | 59 | 47 | 46 | 68 | 46 | 42 | 45 |
| 20 | 9 | 7 | 7 | 6 | 7 | 45 | 71 | 47 | 55 | 46 | 55 | 54 | 53 | 38 | 56 |
| 21 | 10 | 9 | 8 | 10 | 6 | 36 | 55 | 36 | 59 | 47 |  |  |  |  |  |
| 22 | 8 | 5 | 7 | 7 | 6 | 41 | 69 | 39 | 71 | 46 |  |  |  |  |  |
| 23 | 9 | 7 | 7 | 10 | 7 | 43 | 71 | 40 | 77 | 51 |  |  |  |  |  |
| 24 | 8 | 7 | 6 | 7 | 5 | 40 | 83 | 43 | 52 | 45 |  |  |  |  |  |
| 25 | 6 | 8 | 5 | 10 | 8 | 36 | 64 | 40 | 71 | 56 |  |  |  |  |  |
| 26 | 7 | 7 | 6 | 7 | 7 | 34 | 54 | 43 | 65 | 50 |  |  |  |  |  |
| 27 | 7 | 7 | 3 | 8 | 8 | 39 | 35 | 40 | 68 | 47 |  |  |  |  |  |
| 28 | 8 | 9 | 7 | 7 | 5 | 47 | 85 | 39 | 65 | 44 |  |  |  |  |  |
| 29 | 7 | 9 | 5 | 5 | 6 | 42 | 67 | 37 | 75 | 43 |  |  |  |  |  |
| 30 | 9 | 6 | 4 | 9 | 6 | 39 | 77 | 40 | 56 | 49 |  |  |  |  |  |
|  |  |  |  |  |  |  |  |  | 65 |  |  |  |  |  |  |

Note: ctrl means *control group*; the unit of dose is *μL/mL*
