# Supplementary material for: Effects of commercial beverages on the neurobehavioral motility of Caenorhabditis elegans
Source: PeerJ. 2022 Jul 14;10:e13563. doi: 10.7717/peerj.13563 (PMC9288823; doi:10.7717/peerj.13563)
Supplement: Supplemental Information 17 [file peerj-10-13563-s017.docx]

**Table S17--raw data--Neurobehavioral changes of nematodes treated by almond drink**

| **No.** | **body bend** | | | | | **head thrash** | | | | | **pharyngeal pump** | | | | |
| --- | --- | --- | --- | --- | --- | --- | --- | --- | --- | --- | --- | --- | --- | --- | --- |
|  | 500 | 250 | 125 | 62.5 | ctr | 500 | 250 | 125 | 62.5 | ctr | 500 | 250 | 125 | 62.5 | ctr |
| 1 | 8 | 7 | 6 | 11 | 4 | 30 | 48 | 62 | 53 | 22 | 59 | 55 | 51 | 41 | 54 |
| 2 | 4 | 6 | 6 | 6 | 4 | 30 | 42 | 50 | 50 | 59 | 57 | 49 | 28 | 63 | 27 |
| 3 | 5 | 12 | 5 | 10 | 3 | 42 | 28 | 49 | 41 | 42 | 42 | 48 | 47 | 49 | 45 |
| 4 | 6 | 9 | 7 | 16 | 6 | 50 | 39 | 44 | 58 | 38 | 42 | 46 | 46 | 62 | 44 |
| 5 | 5 | 11 | 6 | 10 | 4 | 28 | 41 | 59 | 58 | 49 | 29 | 54 | 36 | 59 | 40 |
| 6 | 7 | 6 | 5 | 11 | 5 | 29 | 29 | 37 | 54 | 51 | 64 | 43 | 58 | 62 | 45 |
| 7 | 8 | 7 | 10 | 12 | 5 | 60 | 54 | 35 | 53 | 53 | 40 | 48 | 55 | 43 | 51 |
| 8 | 8 | 5 | 8 | 11 | 4 | 50 | 45 | 52 | 41 | 37 | 68 | 45 | 54 | 52 | 42 |
| 9 | 5 | 10 | 6 | 11 | 7 | 49 | 42 | 48 | 55 | 45 | 66 | 56 | 29 | 63 | 53 |
| 10 | 7 | 18 | 8 | 15 | 6 | 42 | 41 | 51 | 45 | 47 | 36 | 54 | 48 | 34 | 40 |
| 11 | 12 | 14 | 11 | 7 | 9 | 54 | 56 | 49 | 50 | 57 | 66 | 60 | 32 | 33 | 43 |
| 12 | 10 | 13 | 8 | 15 | 6 | 58 | 49 | 45 | 64 | 58 | 62 | 62 | 47 | 22 | 37 |
| 13 | 11 | 7 | 7 | 9 | 7 | 47 | 56 | 47 | 48 | 62 | 67 | 59 | 54 | 45 | 46 |
| 14 | 12 | 15 | 9 | 3 | 8 | 28 | 46 | 50 | 33 | 56 | 56 | 61 | 44 | 43 | 39 |
| 15 | 13 | 10 | 7 | 14 | 6 | 40 | 45 | 60 | 51 | 47 | 30 | 39 | 54 | 46 | 49 |
| 16 | 7 | 7 | 9 | 9 | 8 | 56 | 50 | 59 | 34 | 46 | 67 | 50 | 55 | 34 | 37 |
| 17 | 8 | 12 | 8 | 11 | 7 | 40 | 42 | 54 | 47 | 48 | 64 | 54 | 66 | 46 | 33 |
| 18 | 14 | 14 | 9 | 7 | 3 | 50 | 59 | 52 | 23 | 54 | 54 | 62 | 49 | 34 | 34 |
| 19 | 10 | 6 | 10 | 6 | 6 | 46 | 36 | 36 | 20 | 43 | 32 | 51 | 52 | 46 | 33 |
| 20 | 9 | 10 | 9 | 7 | 4 | 61 | 52 | 58 | 45 | 45 | 35 | 42 | 40 | 55 | 36 |
| 21 | 8 | 14 | 8 | 12 | 7 | 69 | 64 | 60 | 50 | 43 | 56 |  |  | 50 |  |
| 22 | 9 | 10 | 8 | 4 | 6 | 47 | 45 | 45 | 51 | 38 | 39 |  |  | 45 |  |
| 23 | 6 | 12 | 12 | 11 | 6 | 56 | 46 | 26 | 31 | 40 |  |  |  |  |  |
| 24 | 11 | 4 | 7 | 10 | 6 | 53 | 39 | 59 | 18 | 48 |  |  |  |  |  |
| 25 | 8 | 15 | 6 | 6 | 4 | 49 | 36 | 46 | 23 | 56 |  |  |  |  |  |
| 26 | 9 | 8 | 10 | 10 | 4 | 45 | 48 | 48 | 54 | 49 |  |  |  |  |  |
| 27 | 6 | 6 | 9 | 9 | 3 | 40 | 46 | 56 | 70 | 34 |  |  |  |  |  |
| 28 | 7 | 7 | 7 | 9 | 6 | 48 | 47 | 36 | 58 | 34 |  |  |  |  |  |
| 29 | 10 | 10 | 8 | 6 | 7 | 34 | 52 | 62 | 46 | 36 |  |  |  |  |  |
| 30 | 9 | 9 | 9 | 17 | 6 | 53 | 33 | 46 | 40 | 42 |  |  |  |  |  |

Note: ctrl means *control group*; the unit of dose is *μL/mL*
