# Supplementary material for: Effects of commercial beverages on the neurobehavioral motility of Caenorhabditis elegans
Source: PeerJ. 2022 Jul 14;10:e13563. doi: 10.7717/peerj.13563 (PMC9288823; doi:10.7717/peerj.13563)
Supplement: Supplemental Information 18 [file peerj-10-13563-s018.docx]

**Table S18--raw data--Neurobehavioral changes of nematodes treated by coconut drink**

| **No.** | **body bend** | | | | | **head thrash** | | | | | **pharyngeal pump** | | | | |
| --- | --- | --- | --- | --- | --- | --- | --- | --- | --- | --- | --- | --- | --- | --- | --- |
|  | 500 | 250 | 125 | 62.5 | ctr | 500 | 250 | 125 | 62.5 | ctr | 500 | 250 | 125 | 62.5 | ctr |
| 1 | 7 | 5 | 12 | 8 | 4 | 36 | 40 | 46 | 68 | 22 | 51 | 49 | 60 | 41 | 54 |
| 2 | 8 | 7 | 10 | 7 | 4 | 34 | 39 | 44 | 60 | 59 | 52 | 42 | 41 | 63 | 27 |
| 3 | 9 | 6 | 6 | 10 | 3 | 40 | 40 | 36 | 62 | 42 | 40 | 56 | 41 | 54 | 45 |
| 4 | 8 | 7 | 8 | 7 | 6 | 34 | 30 | 42 | 64 | 38 | 44 | 54 | 58 | 51 | 44 |
| 5 | 7 | 5 | 13 | 6 | 4 | 30 | 40 | 38 | 68 | 49 | 27 | 57 | 42 | 51 | 40 |
| 6 | 5 | 7 | 13 | 7 | 5 | 32 | 34 | 36 | 70 | 51 | 62 | 31 | 53 | 56 | 45 |
| 7 | 7 | 8 | 10 | 9 | 5 | 34 | 40 | 44 | 68 | 53 | 40 | 40 | 43 | 52 | 51 |
| 8 | 5 | 6 | 10 | 7 | 4 | 32 | 30 | 40 | 70 | 37 | 42 | 58 | 46 | 50 | 42 |
| 9 | 6 | 10 | 17 | 8 | 7 | 30 | 38 | 39 | 66 | 45 | 56 | 15 | 43 | 62 | 53 |
| 10 | 7 | 9 | 8 | 5 | 6 | 28 | 32 | 43 | 64 | 47 | 59 | 42 | 41 | 67 | 40 |
| 11 | 5 | 12 | 18 | 6 | 9 | 26 | 40 | 38 | 68 | 57 | 46 | 51 | 47 | 63 | 43 |
| 12 | 5 | 14 | 8 | 7 | 6 | 30 | 37 | 50 | 62 | 58 | 54 | 50 | 18 | 52 | 37 |
| 13 | 6 | 10 | 14 | 6 | 7 | 32 | 40 | 50 | 76 | 62 | 50 | 50 | 53 | 45 | 46 |
| 14 | 7 | 6 | 17 | 5 | 8 | 28 | 42 | 47 | 62 | 56 | 44 | 42 | 50 | 58 | 39 |
| 15 | 6 | 13 | 16 | 6 | 6 | 30 | 40 | 40 | 64 | 47 | 52 | 43 | 31 | 40 | 49 |
| 16 | 6 | 12 | 12 | 6 | 8 | 30 | 44 | 50 | 66 | 46 | 45 | 31 | 54 | 52 | 37 |
| 17 | 5 | 7 | 10 | 7 | 7 | 26 | 38 | 40 | 60 | 48 | 32 | 46 | 49 | 35 | 33 |
| 18 | 6 | 13 | 13 | 8 | 3 | 28 | 32 | 47 | 60 | 54 | 57 | 46 | 60 | 59 | 34 |
| 19 | 7 | 7 | 8 | 8 | 6 | 34 | 46 | 49 | 58 | 43 | 33 | 57 | 46 | 52 | 33 |
| 20 | 7 | 6 | 10 | 8 | 4 | 28 | 38 | 51 | 56 | 45 | 36 | 40 | 59 | 59 | 36 |
| 21 | 4 | 11 | 12 | 11 | 7 | 44 | 42 | 44 | 39 | 43 |  | 58 |  |  |  |
| 22 | 5 | 7 | 10 | 13 | 6 | 36 | 46 | 44 | 47 | 38 |  | 40 |  |  |  |
| 23 | 5 | 9 | 8 | 11 | 6 | 32 | 46 | 38 | 44 | 40 |  |  |  |  |  |
| 24 | 6 | 9 | 9 | 12 | 6 | 34 | 44 | 44 | 39 | 48 |  |  |  |  |  |
| 25 | 5 | 8 | 9 | 12 | 4 | 40 | 50 | 46 | 40 | 56 |  |  |  |  |  |
| 26 | 7 | 6 | 8 | 8 | 4 | 42 | 42 | 40 | 45 | 49 |  |  |  |  |  |
| 27 | 8 | 7 | 11 | 9 | 3 | 46 | 48 | 42 | 43 | 34 |  |  |  |  |  |
| 28 | 5 | 8 | 9 | 10 | 6 | 38 | 42 | 35 | 48 | 34 |  |  |  |  |  |
| 29 |  | 10 | 8 | 8 | 7 | 40 | 36 | 36 | 37 | 36 |  |  |  |  |  |
| 30 |  | 7 | 8 | 5 | 6 | 44 | 44 | 49 | 36 | 42 |  |  |  |  |  |

Note: ctrl means *control group*; the unit of dose is *μL/mL*
