# Supplementary material for: Effects of commercial beverages on the neurobehavioral motility of Caenorhabditis elegans
Source: PeerJ. 2022 Jul 14;10:e13563. doi: 10.7717/peerj.13563 (PMC9288823; doi:10.7717/peerj.13563)
Supplement: Supplemental Information 19 [file peerj-10-13563-s019.docx]

**Table S19--raw data--Neurobehavioral changes of nematodes treated by milk tea beverage**

| **No.** | **body bend** | | | | | **head thrash** | | | | | **pharyngeal pump** | | | | |
| --- | --- | --- | --- | --- | --- | --- | --- | --- | --- | --- | --- | --- | --- | --- | --- |
|  | 500 | 250 | 125 | 62.5 | ctr | 500 | 250 | 125 | 62.5 | ctr | 500 | 250 | 125 | 62.5 | ctr |
| 1 | 7 | 7 | 4 | 6 | 4 | 61 | 52 | 57 | 55 | 22 | 56 | 48 | 63 | 50 | 54 |
| 2 | 4 | 7 | 4 | 4 | 4 | 56 | 54 | 45 | 52 | 59 | 43 | 42 | 69 | 59 | 27 |
| 3 | 5 | 6 | 5 | 5 | 3 | 55 | 56 | 50 | 48 | 42 | 53 | 43 | 55 | 53 | 45 |
| 4 | 5 | 6 | 5 | 7 | 6 | 48 | 62 | 33 | 46 | 38 | 42 | 58 | 56 | 50 | 44 |
| 5 | 4 | 4 | 6 | 6 | 4 | 57 | 64 | 54 | 56 | 49 | 46 | 39 | 44 | 66 | 40 |
| 6 | 5 | 5 | 4 | 5 | 5 | 56 | 62 | 42 | 41 | 51 | 40 | 53 | 62 | 44 | 45 |
| 7 | 9 | 4 | 5 | 5 | 5 | 55 | 56 | 46 | 44 | 53 | 57 | 64 | 70 | 65 | 51 |
| 8 | 3 | 5 | 7 | 7 | 4 | 50 | 58 | 50 | 47 | 37 | 41 | 49 | 54 | 76 | 42 |
| 9 | 4 | 4 | 5 | 8 | 7 | 56 | 56 | 48 | 48 | 45 | 43 | 53 | 66 | 72 | 53 |
| 10 | 9 | 6 | 4 | 6 | 6 | 53 | 54 | 45 | 52 | 47 | 45 | 61 | 64 | 49 | 40 |
| 11 | 3 | 11 | 6 | 5 | 9 | 57 | 52 | 67 | 50 | 57 | 40 | 60 | 56 | 49 | 43 |
| 12 | 7 | 10 | 7 | 7 | 6 | 59 | 58 | 52 | 57 | 58 | 46 | 62 | 49 | 62 | 37 |
| 13 | 6 | 15 | 7 | 6 | 7 | 58 | 72 | 58 | 54 | 62 | 44 | 51 | 50 | 52 | 46 |
| 14 | 4 | 5 | 5 | 7 | 8 | 56 | 70 | 59 | 62 | 56 | 65 | 52 | 56 | 65 | 39 |
| 15 | 8 | 4 | 7 | 9 | 6 | 57 | 60 | 60 | 61 | 47 | 63 | 49 | 57 | 43 | 49 |
| 16 | 9 | 9 | 10 | 6 | 8 | 50 | 56 | 54 | 58 | 46 | 59 | 63 | 72 | 66 | 37 |
| 17 | 9 | 5 | 6 | 7 | 7 | 47 | 60 | 54 | 48 | 48 | 46 | 55 | 68 | 63 | 33 |
| 18 | 6 | 5 | 4 | 4 | 3 | 51 | 72 | 43 | 54 | 54 | 42 | 48 | 71 | 53 | 34 |
| 19 | 5 | 3 | 9 | 6 | 6 | 53 | 66 | 46 | 53 | 43 | 51 | 45 | 64 | 62 | 33 |
| 20 | 6 | 7 | 4 | 9 | 4 | 57 | 62 | 51 | 47 | 45 | 52 | 55 | 60 | 58 | 36 |
| 21 | 14 | 10 | 7 | 10 | 7 | 61 | 78 | 51 | 54 | 43 |  |  |  |  |  |
| 22 | 7 | 8 | 4 | 6 | 6 | 56 | 62 | 54 | 56 | 38 |  |  |  |  |  |
| 23 | 7 | 11 | 5 | 7 | 6 | 55 | 66 | 54 | 49 | 40 |  |  |  |  |  |
| 24 | 6 | 11 | 6 | 6 | 6 | 48 | 56 | 53 | 42 | 48 |  |  |  |  |  |
| 25 | 5 | 5 | 6 | 4 | 4 | 57 | 64 | 45 | 39 | 56 |  |  |  |  |  |
| 26 | 6 | 6 | 7 | 5 | 4 | 56 | 70 | 43 | 41 | 49 |  |  |  |  |  |
| 27 | 9 | 4 | 6 | 5 | 3 | 55 | 62 | 50 | 49 | 34 |  |  |  |  |  |
| 28 | 5 | 9 | 9 | 5 | 6 | 50 | 68 | 48 | 48 | 34 |  |  |  |  |  |
| 29 | 10 | 9 | 6 | 4 | 7 | 56 | 68 | 55 | 44 | 36 |  |  |  |  |  |
| 30 | 5 | 8 | 4 | 6 | 6 | 53 | 60 | 52 | 43 | 42 |  |  |  |  |  |

Note: ctrl means *control group*; the unit of dose is *μL/mL*
