# Supplementary material for: Effects of commercial beverages on the neurobehavioral motility of Caenorhabditis elegans
Source: PeerJ. 2022 Jul 14;10:e13563. doi: 10.7717/peerj.13563 (PMC9288823; doi:10.7717/peerj.13563)
Supplement: Supplemental Information 20 [file peerj-10-13563-s020.docx]

**Table S20--raw data--Neurobehavioral changes of nematodes treated by**

**prepared milk beverage A**

| **No.** | **body bend** | | | | | **head thrash** | | | | | **pharyngeal pump** | | | | |
| --- | --- | --- | --- | --- | --- | --- | --- | --- | --- | --- | --- | --- | --- | --- | --- |
|  | 500 | 250 | 125 | 62.5 | ctr | 500 | 250 | 125 | 62.5 | ctr | 500 | 250 | 125 | 62.5 | ctr |
| 1 | 9 | 8 | 10 | 11 | 6 | 70 | 82 | 72 | 82 | 54 | 51 | 61 | 57 | 64 | 61 |
| 2 | 6 | 10 | 10 | 8 | 6 | 55 | 100 | 106 | 86 | 56 | 30 | 60 | 55 | 39 | 33 |
| 3 | 7 | 12 | 9 | 10 | 5 | 56 | 86 | 86 | 86 | 54 | 53 | 48 | 56 | 57 | 56 |
| 4 | 8 | 10 | 12 | 9 | 5 | 54 | 88 | 96 | 80 | 50 | 54 | 52 | 57 | 58 | 57 |
| 5 | 7 | 8 | 9 | 9 | 6 | 56 | 98 | 80 | 94 | 46 | 51 | 59 | 50 | 57 | 62 |
| 6 | 7 | 10 | 11 | 11 | 6 | 70 | 90 | 78 | 100 | 54 | 59 | 54 | 42 | 50 | 56 |
| 7 | 6 | 9 | 9 | 8 | 4 | 66 | 88 | 92 | 80 | 60 | 63 | 61 | 50 | 72 | 51 |
| 8 | 6 | 12 | 8 | 9 | 5 | 50 | 100 | 96 | 78 | 64 | 55 | 49 | 51 | 62 | 62 |
| 9 | 8 | 11 | 13 | 7 | 4 | 88 | 102 | 78 | 82 | 58 | 63 | 57 | 44 | 59 | 48 |
| 10 | 8 | 12 | 13 | 10 | 6 | 59 | 96 | 84 | 76 | 62 | 57 | 67 | 55 | 65 | 38 |
| 11 | 6 | 8 | 9 | 10 | 5 | 96 | 88 | 90 | 106 | 62 | 52 | 66 | 48 | 62 | 38 |
| 12 | 8 | 9 | 10 | 9 | 7 | 72 | 98 | 86 | 98 | 56 | 47 | 51 | 36 | 50 | 37 |
| 13 | 10 | 12 | 9 | 9 | 5 | 68 | 84 | 100 | 98 | 54 | 48 | 52 | 42 | 58 | 59 |
| 14 | 8 | 9 | 10 | 10 | 6 | 90 | 96 | 93 | 100 | 64 | 61 | 57 | 56 | 50 | 57 |
| 15 | 8 | 10 | 8 | 9 | 6 | 90 | 80 | 84 | 86 | 60 | 40 | 55 | 54 | 62 | 50 |
| 16 | 9 | 9 | 9 | 12 | 6 | 96 | 74 | 86 | 96 | 60 | 46 | 47 | 60 | 53 | 49 |
| 17 | 7 | 11 | 11 | 11 | 5 | 94 | 72 | 98 | 80 | 64 | 55 | 46 | 67 | 48 | 33 |
| 18 | 9 | 12 | 13 | 12 | 6 | 86 | 94 | 94 | 76 | 62 | 48 | 57 | 69 | 47 | 56 |
| 19 | 8 | 13 | 11 | 13 | 6 | 80 | 86 | 106 | 86 | 58 | 63 | 66 | 54 | 56 | 60 |
| 20 | 7 | 10 | 8 | 10 | 5 | 100 | 80 | 100 | 80 | 64 | 55 | 50 | 44 | 65 | 51 |
| 21 | 11 | 9 | 10 | 9 | 5 | 108 | 78 | 98 | 100 | 60 |  |  |  |  |  |
| 22 | 7 | 7 | 10 | 10 | 4 | 94 | 92 | 94 | 92 | 58 |  |  |  |  |  |
| 23 | 9 | 10 | 14 | 11 | 7 | 98 | 76 | 84 | 104 | 54 |  |  |  |  |  |
| 24 | 10 | 11 | 9 | 11 | 7 | 80 | 92 | 90 | 96 | 52 |  |  |  |  |  |
| 25 | 10 | 12 | 12 | 10 | 6 | 104 | 90 | 88 | 98 | 64 |  |  |  |  |  |
| 26 | 9 | 12 | 15 | 9 | 5 | 82 | 84 | 102 | 98 | 66 |  |  |  |  |  |
| 27 | 9 | 8 | 10 | 10 | 4 | 88 | 80 | 108 | 86 | 60 |  |  |  |  |  |
| 28 | 9 | 9 | 9 | 8 | 6 | 96 | 84 | 100 | 98 | 56 |  |  |  |  |  |
| 29 | 12 | 13 | 12 | 11 | 5 | 80 | 80 | 86 | 106 | 68 |  |  |  |  |  |
| 30 | 7 | 11 | 11 | 12 | 5 | 88 | 94 | 104 | 100 | 58 |  |  |  |  |  |

Note: ctrl means *control group*; the unit of dose is *μL/mL*
