# Supplementary material for: Effects of commercial beverages on the neurobehavioral motility of Caenorhabditis elegans
Source: PeerJ. 2022 Jul 14;10:e13563. doi: 10.7717/peerj.13563 (PMC9288823; doi:10.7717/peerj.13563)
Supplement: Supplemental Information 21 [file peerj-10-13563-s021.docx]

**Table S21--raw data--Neurobehavioral changes of nematodes treated by**

**prepared milk beverage B**

| **No.** | **body bend** | | | | | **head thrash** | | | | | **pharyngeal pump** | | | | |
| --- | --- | --- | --- | --- | --- | --- | --- | --- | --- | --- | --- | --- | --- | --- | --- |
|  | 500 | 250 | 125 | 62.5 | ctr | 500 | 250 | 125 | 62.5 | ctr | 500 | 250 | 125 | 62.5 | ctr |
| 1 | 10 | 11 | 6 | 5 | 6 | 72 | 96 | 44 | 43 | 54 | 67 | 48 | 50 | 60 | 61 |
| 2 | 9 | 14 | 7 | 7 | 6 | 64 | 84 | 49 | 38 | 56 | 71 | 41 | 51 | 72 | 33 |
| 3 | 9 | 10 | 5 | 6 | 5 | 73 | 88 | 53 | 53 | 54 | 42 | 65 | 34 | 67 | 56 |
| 4 | 8 | 12 | 6 | 5 | 5 | 90 | 96 | 47 | 34 | 50 | 48 | 60 | 50 | 58 | 57 |
| 5 | 4 | 13 | 5 | 3 | 6 | 70 | 102 | 47 | 64 | 46 | 61 | 60 | 51 | 62 | 62 |
| 6 | 8 | 10 | 8 | 4 | 6 | 92 | 70 | 39 | 56 | 54 | 42 | 54 | 48 | 60 | 56 |
| 7 | 10 | 9 | 6 | 6 | 4 | 40 | 100 | 42 | 47 | 60 | 61 | 54 | 53 | 65 | 51 |
| 8 | 10 | 6 | 7 | 4 | 5 | 80 | 99 | 53 | 53 | 64 | 67 | 51 | 65 | 49 | 62 |
| 9 | 8 | 7 | 5 | 7 | 4 | 89 | 92 | 57 | 38 | 58 | 57 | 55 | 64 | 55 | 48 |
| 10 | 7 | 10 | 4 | 5 | 6 | 97 | 85 | 49 | 44 | 62 | 66 | 58 | 65 | 66 | 38 |
| 11 | 10 | 11 | 6 | 5 | 5 | 90 | 94 | 47 | 47 | 62 | 59 | 45 | 63 | 64 | 38 |
| 12 | 11 | 11 | 4 | 5 | 7 | 81 | 96 | 52 | 54 | 56 | 73 | 51 | 55 | 58 | 37 |
| 13 | 9 | 11 | 7 | 6 | 5 | 88 | 80 | 48 | 42 | 54 | 57 | 51 | 63 | 46 | 59 |
| 14 | 8 | 9 | 4 | 6 | 6 | 60 | 72 | 38 | 51 | 64 | 53 | 62 | 56 | 65 | 57 |
| 15 | 9 | 8 | 5 | 8 | 6 | 82 | 80 | 37 | 42 | 60 | 59 | 57 | 57 | 63 | 50 |
| 16 | 9 | 10 | 6 | 7 | 6 | 77 | 96 | 60 | 41 | 60 | 57 | 55 | 59 | 59 | 49 |
| 17 | 8 | 12 | 5 | 5 | 5 | 96 | 94 | 44 | 52 | 64 | 50 | 54 | 58 | 54 | 33 |
| 18 | 7 | 9 | 7 | 5 | 6 | 92 | 90 | 37 | 41 | 62 | 59 | 45 | 55 | 63 | 56 |
| 19 | 9 | 10 | 6 | 4 | 6 | 54 | 96 | 51 | 48 | 58 | 62 | 55 | 69 | 54 | 60 |
| 20 | 9 | 8 | 4 | 6 | 5 | 80 | 88 | 53 | 50 | 64 | 47 | 49 | 62 | 55 | 51 |
| 21 | 9 | 13 | 4 | 7 | 5 | 82 | 86 | 58 | 48 | 60 |  |  |  |  |  |
| 22 | 12 | 12 | 7 | 5 | 4 | 90 | 85 | 53 | 44 | 58 |  |  |  |  |  |
| 23 | 10 | 10 | 5 | 6 | 7 | 79 | 69 | 50 | 39 | 54 |  |  |  |  |  |
| 24 | 12 | 14 | 6 | 6 | 7 | 74 | 76 | 48 | 44 | 52 |  |  |  |  |  |
| 25 | 10 | 12 | 5 | 3 | 6 | 82 | 90 | 52 | 49 | 64 |  |  |  |  |  |
| 26 | 10 | 5 | 5 | 4 | 5 | 92 | 94 | 50 | 42 | 66 |  |  |  |  |  |
| 27 | 8 | 6 | 5 | 4 | 4 | 84 | 76 | 47 | 49 | 60 |  |  |  |  |  |
| 28 | 10 | 8 | 4 | 3 | 6 | 88 | 84 | 49 | 53 | 56 |  |  |  |  |  |
| 29 | 11 | 11 | 7 | 5 | 5 | 96 | 94 | 57 | 46 | 68 |  |  |  |  |  |
| 30 | 9 | 8 | 3 | 3 | 5 | 88 | 64 | 49 | 49 | 58 |  |  |  |  |  |

Note: ctrl means *control group*; the unit of dose is *μL/mL*
