# Supplementary material for: Effects of commercial beverages on the neurobehavioral motility of Caenorhabditis elegans
Source: PeerJ. 2022 Jul 14;10:e13563. doi: 10.7717/peerj.13563 (PMC9288823; doi:10.7717/peerj.13563)
Supplement: Supplemental Information 22 [file peerj-10-13563-s022.docx]

**Table S22--raw data--Neurobehavioral changes of nematodes treated by**

**prepared milk beverage C**

| **No.** | **body bend** | | | | | **head thrash** | | | | | **pharyngeal pump** | | | | |
| --- | --- | --- | --- | --- | --- | --- | --- | --- | --- | --- | --- | --- | --- | --- | --- |
|  | 500 | 250 | 125 | 62.5 | ctr | 500 | 250 | 125 | 62.5 | ctr | 500 | 250 | 125 | 62.5 | ctr |
| 1 | 2 | 7 | 7 | 6 | 8 | 60 | 46 | 56 | 58 | 50 | 40 | 52 | 47 | 66 | 62 |
| 2 | 3 | 5 | 6 | 4 | 5 | 44 | 48 | 48 | 64 | 46 | 45 | 46 | 66 | 63 | 0 |
| 3 | 5 | 5 | 4 | 5 | 8 | 52 | 52 | 54 | 66 | 90 | 48 | 42 | 54 | 62 | 37 |
| 4 | 3 | 4 | 5 | 6 | 8 | 56 | 52 | 48 | 70 | 91 | 39 | 45 | 48 | 63 | 77 |
| 5 | 7 | 4 | 5 | 5 | 9 | 50 | 44 | 60 | 58 | 66 | 51 | 54 | 47 | 71 | 69 |
| 6 | 4 | 5 | 6 | 6 | 8 | 48 | 52 | 60 | 60 | 88 | 56 | 56 | 49 | 67 | 53 |
| 7 | 5 | 4 | 5 | 6 | 9 | 52 | 58 | 64 | 74 | 74 | 58 | 49 | 69 | 52 | 36 |
| 8 | 4 | 5 | 5 | 7 | 7 | 60 | 56 | 58 | 78 | 88 | 51 | 48 | 42 | 29 | 65 |
| 9 | 5 | 5 | 4 | 5 | 6 | 62 | 50 | 66 | 78 | 84 | 50 | 49 | 47 | 44 | 55 |
| 10 | 6 | 4 | 5 | 6 | 8 | 56 | 66 | 54 | 68 | 44 | 54 | 55 | 50 | 54 | 76 |
| 11 | 7 | 4 | 4 | 6 | 6 | 60 | 64 | 62 | 58 | 74 | 51 | 54 | 42 | 31 | 74 |
| 12 | 4 | 5 | 6 | 7 | 7 | 62 | 58 | 58 | 54 | 80 | 45 | 50 | 54 | 51 | 42 |
| 13 | 5 | 6 | 5 | 5 | 5 | 58 | 64 | 60 | 78 | 78 | 52 | 68 | 48 | 62 | 63 |
| 14 | 6 | 4 | 6 | 8 | 9 | 62 | 66 | 64 | 66 | 80 | 55 | 63 | 56 | 57 | 32 |
| 15 | 5 | 7 | 5 | 4 | 6 | 44 | 50 | 52 | 66 | 76 | 54 | 65 | 49 | 64 | 77 |
| 16 | 6 | 5 | 4 | 5 | 7 | 60 | 58 | 66 | 58 | 54 | 52 | 62 | 47 | 56 | 45 |
| 17 | 6 | 6 | 4 | 6 | 7 | 58 | 62 | 64 | 60 | 72 | 55 | 59 | 46 | 51 | 71 |
| 18 | 6 | 5 | 6 | 7 | 6 | 56 | 58 | 58 | 58 | 84 | 50 | 58 | 46 | 52 | 40 |
| 19 | 6 | 5 | 5 | 7 | 5 | 64 | 56 | 66 | 78 | 58 | 48 | 53 | 45 | 40 | 63 |
| 20 | 4 | 4 | 5 | 6 | 5 | 58 | 54 | 48 | 68 | 54 | 51 | 45 | 49 | 58 | 35 |
| 21 | 10 | 6 | 9 | 7 | 8 | 52 | 64 | 64 | 62 | 60 | 44 |  |  |  |  |
| 22 | 5 | 7 | 9 | 6 | 6 | 48 | 62 | 52 | 68 | 86 |  |  |  |  |  |
| 23 | 6 | 8 | 8 | 8 | 5 | 64 | 54 | 60 | 72 | 78 |  |  |  |  |  |
| 24 | 5 | 5 | 7 | 8 | 7 | 56 | 58 | 56 | 56 | 45 |  |  |  |  |  |
| 25 | 6 | 6 | 10 | 5 | 5 | 52 | 66 | 72 | 48 | 84 |  |  |  |  |  |
| 26 | 5 | 5 | 8 | 5 | 5 | 54 | 68 | 60 | 48 | 72 |  |  |  |  |  |
| 27 | 3 | 7 | 4 | 6 | 6 | 56 | 52 | 66 | 68 | 68 |  |  |  |  |  |
| 28 | 3 | 5 | 6 | 9 | 4 | 64 | 66 | 70 | 58 | 74 |  |  |  |  |  |
| 29 | 5 | 6 | 5 | 9 | 9 | 58 | 64 | 60 | 72 | 88 |  |  |  |  |  |
| 30 | 4 | 6 | 7 | 5 | 7 | 62 | 58 | 58 | 58 | 64 |  |  |  |  |  |

Note: ctrl means *control group*; the unit of dose is *μL/mL*
