# Supplementary material for: Effects of commercial beverages on the neurobehavioral motility of Caenorhabditis elegans
Source: PeerJ. 2022 Jul 14;10:e13563. doi: 10.7717/peerj.13563 (PMC9288823; doi:10.7717/peerj.13563)
Supplement: Supplemental Information 23 [file peerj-10-13563-s023.docx]

**Table S23--raw data--Neurobehavioral changes of nematodes treated by**

**prepared milk beverage D**

| **No.** | **body bend** | | | | | **head thrash** | | | | | **pharyngeal pump** | | | | |
| --- | --- | --- | --- | --- | --- | --- | --- | --- | --- | --- | --- | --- | --- | --- | --- |
|  | 500 | 250 | 125 | 62.5 | ctr | 500 | 250 | 125 | 62.5 | ctr | 500 | 250 | 125 | 62.5 | ctr |
| 1 | 6 | 12 | 8 | 5 | 8 | 46 | 86 | 43 | 64 | 50 | 55 | 55 | 10 | 55 | 62 |
| 2 | 4 | 8 | 9 | 6 | 5 | 42 | 78 | 55 | 44 | 46 | 12 | 50 | 34 | 26 | 0 |
| 3 | 6 | 12 | 10 | 7 | 8 | 44 | 38 | 52 | 68 | 90 | 65 | 39 | 33 | 44 | 37 |
| 4 | 7 | 9 | 8 | 7 | 8 | 42 | 76 | 54 | 72 | 91 | 21 | 38 | 33 | 38 | 77 |
| 5 | 10 | 13 | 7 | 9 | 9 | 54 | 66 | 59 | 78 | 66 | 57 | 49 | 49 | 56 | 69 |
| 6 | 6 | 8 | 6 | 6 | 8 | 53 | 74 | 44 | 54 | 88 | 43 | 56 | 61 | 46 | 53 |
| 7 | 7 | 10 | 5 | 5 | 9 | 51 | 80 | 57 | 58 | 74 | 46 | 44 | 54 | 40 | 36 |
| 8 | 5 | 9 | 3 | 6 | 7 | 52 | 90 | 49 | 69 | 88 | 35 | 41 | 53 | 51 | 65 |
| 9 | 5 | 9 | 7 | 6 | 6 | 47 | 58 | 57 | 83 | 84 | 42 | 51 | 42 | 58 | 55 |
| 10 | 4 | 8 | 5 | 7 | 8 | 49 | 72 | 53 | 72 | 44 | 57 | 58 | 51 | 31 | 76 |
| 11 | 5 | 11 | 12 | 9 | 6 | 63 | 54 | 65 | 60 | 74 | 59 | 46 | 36 | 28 | 74 |
| 12 | 8 | 9 | 8 | 6 | 7 | 62 | 56 | 50 | 64 | 80 | 46 | 49 | 51 | 42 | 42 |
| 13 | 5 | 10 | 8 | 9 | 5 | 60 | 78 | 64 | 48 | 78 | 38 | 54 | 43 | 30 | 63 |
| 14 | 6 | 9 | 7 | 9 | 9 | 57 | 84 | 63 | 50 | 80 | 55 | 43 | 43 | 8 | 32 |
| 15 | 4 | 8 | 6 | 8 | 6 | 61 | 74 | 64 | 46 | 76 | 40 | 53 | 49 | 49 | 77 |
| 16 | 3 | 9 | 11 | 8 | 7 | 55 | 70 | 67 | 52 | 54 | 54 | 36 | 26 | 46 | 45 |
| 17 | 8 | 12 | 5 | 12 | 7 | 73 | 80 | 69 | 54 | 72 | 48 | 29 | 53 | 40 | 71 |
| 18 | 4 | 6 | 7 | 6 | 6 | 63 | 54 | 64 | 42 | 84 | 63 | 55 | 38 | 30 | 40 |
| 19 | 5 | 9 | 7 | 10 | 5 | 50 | 84 | 67 | 56 | 58 | 61 | 50 | 36 | 58 | 63 |
| 20 | 4 | 10 | 4 | 11 | 5 | 56 | 76 | 62 | 44 | 54 | 43 | 56 | 48 | 42 | 35 |
| 21 | 6 | 13 | 8 | 7 | 8 | 60 | 94 | 48 | 68 | 60 |  |  |  |  |  |
| 22 | 4 | 8 | 10 | 9 | 6 | 55 | 82 | 62 | 70 | 86 |  |  |  |  |  |
| 23 | 6 | 10 | 9 | 4 | 5 | 60 | 84 | 67 | 60 | 78 |  |  |  |  |  |
| 24 | 8 | 10 | 7 | 5 | 7 | 55 | 88 | 57 | 84 | 45 |  |  |  |  |  |
| 25 | 4 | 9 | 6 | 7 | 5 | 50 | 80 | 60 | 68 | 84 |  |  |  |  |  |
| 26 | 5 | 11 | 7 | 7 | 5 | 63 | 90 | 62 | 60 | 72 |  |  |  |  |  |
| 27 | 5 | 9 | 7 | 7 | 6 | 70 | 92 | 48 | 68 | 68 |  |  |  |  |  |
| 28 | 9 | 6 | 11 | 6 | 4 | 49 | 88 | 59 | 80 | 74 |  |  |  |  |  |
| 29 | 10 | 10 | 9 | 7 | 9 | 54 | 62 | 63 | 54 | 88 |  |  |  |  |  |
| 30 | 9 | 11 | 8 | 5 | 7 | 67 | 94 | 64 | 86 | 64 |  |  |  |  |  |

Note:ctrl means control group; the unit of dose is μL/mL
