# Supplementary figures and images for: Effects of commercial beverages on the neurobehavioral motility of Caenorhabditis elegans
Source: PeerJ. 2022 Jul 14;10:e13563. doi: 10.7717/peerj.13563 (PMC9288823; doi:10.7717/peerj.13563)

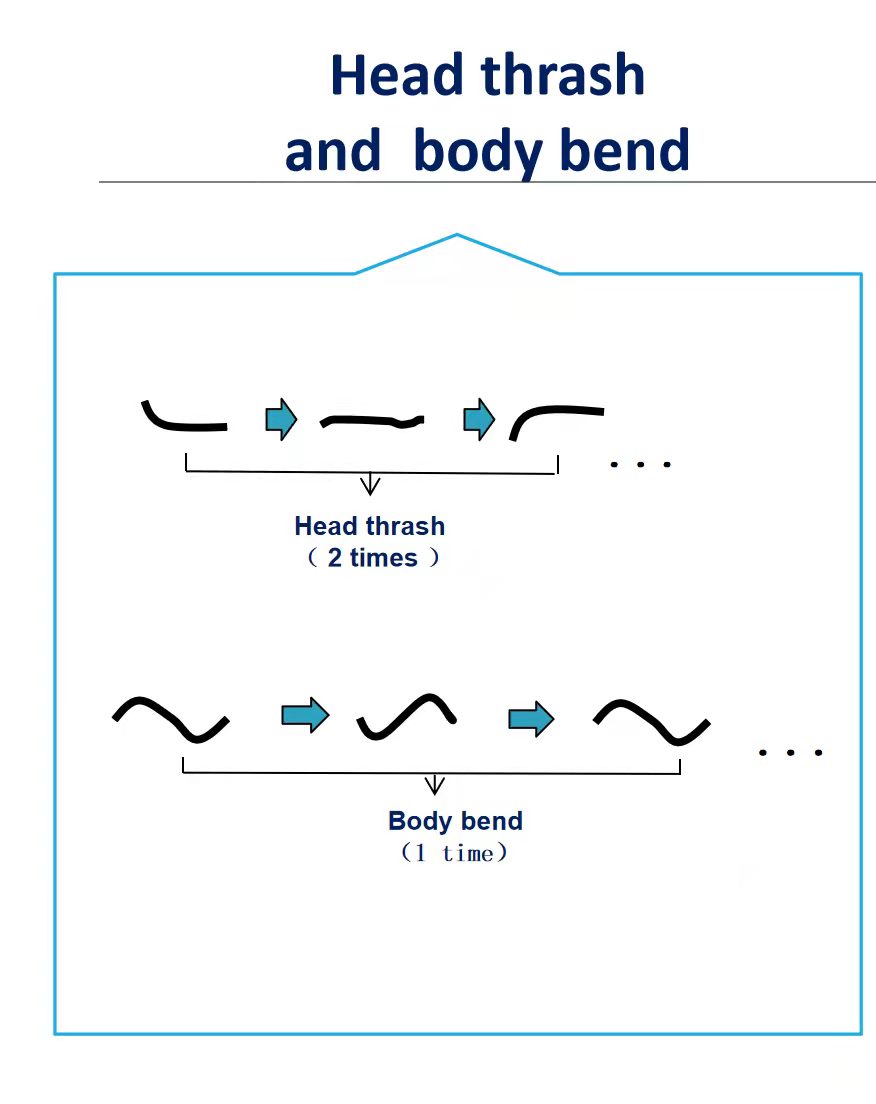

Supplement: Supplemental Information 24 [file peerj-10-13563-s024.jpg]
